# School-based peer education interventions to improve health: a global systematic review of effectiveness

**DOI:** 10.1186/s12889-022-14688-3

**Published:** 2022-12-02

**Authors:** Steven Dodd, Emily Widnall, Abigail Emma Russell, Esther Louise Curtin, Ruth Simmonds, Mark Limmer, Judi Kidger

**Affiliations:** 1 Faculty of Health and Medicine, Lancaster University, Lancaster, UK; 2 Population Health Sciences, University of Bristol, Bristol, UK; 3 College of Medicine and Health, University of Exeter, Exeter, UK; 4 Faculty of Epidemiology and Population Health, London School of Hygiene and Tropical Medicine, London, UK; 5 Mental Health Foundation, London, UK

**Keywords:** Peer education, School-based interventions, Systematic review, School health, Adolescents

## Abstract

**Introduction:**

Peer education, whereby peers (‘peer educators’) teach their other peers (‘peer learners’) about aspects of health is an approach growing in popularity across school contexts, possibly due to adolescents preferring to seek help for health-related concerns from their peers rather than adults or professionals. Peer education interventions cover a wide range of health areas but their overall effectiveness remains unclear. This review aims to summarise the effectiveness of existing peer-led health interventions implemented in schools worldwide.

**Methods:**

Five electronic databases were searched for eligible studies in October 2020. To be included, studies must have evaluated a school-based peer education intervention designed to address the health of students aged 11–18-years-old and include quantitative outcome data to examine effectiveness. The number of interventions were summarised and the impact on improved health knowledge and reductions in health problems or risk-taking behaviours were investigated for each health area separately, the Mixed Methods Appraisal Tool was used to assess quality.

**Results:**

A total of 2125 studies were identified after the initial search and 73 articles were included in the review. The majority of papers evaluated interventions focused on sex education/HIV prevention (*n* = 23), promoting healthy lifestyles (*n* = 17) and alcohol, smoking and substance use (*n* = 16). Papers mainly reported peer learner outcomes (67/73, 91.8%), with only six papers (8.2%) focussing solely on peer educator outcomes and five papers (6.8%) examining both peer learner and peer educator outcomes. Of the 67 papers reporting peer learner outcomes, 35/67 (52.2%) showed evidence of effectiveness, 8/67 (11.9%) showed mixed findings and 24/67 (35.8%) found limited or no evidence of effectiveness. Of the 11 papers reporting peer educator outcomes, 4/11 (36.4%) showed evidence of effectiveness, 2/11 (18.2%) showed mixed findings and 5/11 (45.5%) showed limited or no evidence of effectiveness. Study quality varied greatly with many studies rated as poor quality, mainly due to unrepresentative samples and incomplete data.

**Discussion:**

School-based peer education interventions are implemented worldwide and span a wide range of health areas. A number of interventions appear to demonstrate evidence for effectiveness, suggesting peer education may be a promising strategy for health improvement in schools. Improvement in health-related knowledge was most common with less evidence for positive health behaviour change. In order to quantitatively synthesise the evidence and make more confident conclusions, there is a need for more robust, high-quality evaluations of peer-led interventions using standardised health knowledge and behaviour measures.

**Supplementary Information:**

The online version contains supplementary material available at 10.1186/s12889-022-14688-3.

## Introduction

Ensuring good health and wellbeing amongst school-aged children is a global public health priority and the contribution schools can make to this goal is increasingly recognised [1]. Worldwide, we have seen a rise in peer education interventions over recent decades [2]. For example, a survey in England revealed that 62% of primary and secondary schools had offered a peer-led intervention in 2009 [3]. Peer-led interventions within school settings are popular for many reasons, including the important role peers play within the lives of young people, a perception that this approach involves relatively few resources, and the more even balance of authority than in teacher-led lessons [4]. The use of peer educators for health improvement has also been linked with the importance of peer influence in adolescence [5]. This is a time of increased social development and peer attachments are central to young people’s development, particularly during adolescence [5, 6]. Further, there is evidence that young people are more likely to seek help from informal sources of support such as friends in comparison to adults [7], and of older students being perceived as role models by their younger peers [8]. Benefits are also likely to exist for peer educators themselves, including opportunities to develop confidence and leadership skills, as well as many schools rewarding peer educators with a qualification or endorsement for their participation [9].

Existing peer education interventions cover a wide range of health areas, including mental health, physical health, sexual health, and a general promotion of healthy lifestyles including eating habits and smoking prevention [10–13]. There is also variation in the format or delivery of peer-led interventions including 1:1 peer mentoring, peer buddy initiatives, peer counselling, and peer education [14–17]. This review focuses specifically on peer education, which typically involves the selection and training of ‘peer educators’ or ‘leaders’, who subsequently relay health related information or skills to younger or similar aged students in their school, known as ‘peer learners’ or ‘recipients’.

### Summary of related reviews

The current literature on peer education indicates a mixed evidence base regarding its effectiveness.

Ten previous reviews were found concerning health-related peer education among young people [10, 12, 18–24]. Of these, six concerned sexual health/HIV prevention, two concerned health promotion/education more broadly, one focused on substance abuse and one focused on mental health.

Kim and Free’s review concerning sexual health [21] found no overall effect of peer education on condom use, mixed findings on sexually transmitted infection (STI) prevention, and positive findings regarding improvements in knowledge, attitudes and intentions. Siddiqui et al. [20] reviewed peer education programmes for promoting the sexual and reproductive health of young people in India, revealing large variations in the way peer education is implemented as well as mixed effectiveness findings and limited effects of behaviour relative to knowledge. Maticka-Tyndale and Barnet [22] compiled a review into peer-led interventions to reduce HIV risk among youth using a narrative synthesis, and found that peer interventions led to positive change in knowledge and condom use, and had some success in changing community attitudes and norms, but no significant findings for effects on other sexual behaviours and STI rates. By comparison, Tolli’s review [12] regarding the effectiveness of peer education interventions for HIV prevention found no clear evidence of peer education effectiveness for HIV prevention, adolescent pregnancy prevention or sexual health promotion in young people of member countries of the European Union.

Mellanby et al. [23] reviewed the literature comparing peer-led and adult-led school health education and identified eleven studies. Seven of these studies found peer-led to be more effective for health behaviour change than adult-led and three of these studies found peer-led to me more effective for change in knowledge and attitudes. Harden et al. [24] identified 64 peer-delivered health interventions for young people aged 11 to 24 in any setting (i.e. not restricted to school settings), with only 12 evaluations judged to be methodologically sound. Of these 12, 7 studies (58%) showed a positive effect on at least one behavioural outcome. This review concluded an unclear evidence base for peer-delivered health promotion for young people.

MacArthur et al’s [19] investigation of peer-led interventions to prevent tobacco, alcohol and/or drug use among young people aged 11–21, comprised a meta-analysis, pooling 10 studies on tobacco use, and found lower prevalence of smoking among those receiving the peer-led interventions compared with controls. The authors also found that peer-led interventions were associated with benefit in relation to alcohol use, and three studies suggested an association with lower odds of cannabis use.

A recent systematic review by King and Fazel of 11 school-based peer-led mental health interventions studies revealed mixed effectiveness [10]. Some studies showed significant improvements in peer educator self-esteem and social stress [25], but one study showed an increase in guilt in peer educators [26]. Two studies also found improvements in self-confidence [27], and quality of life in peer learners [28], but one study found an increase in learning stress and decrease in overall mental health scores [26]. The review concluded there is better evidence if benefits for peer educators compared to peer learners. The summary above of previous systematic assessments of the peer education approach reveals a limited evidence base for school-based peer education interventions. Only two reviews were included regarding school-based peer education, one of which occurred over 20 years ago [23], while the other [10] was more narrowly concerned with mental health outcomes.

Despite the widespread use of peer-led interventions, the evidence base across all health areas still remains limited and little is known regarding their overall effectiveness in terms of changing behaviours or increasing health-related knowledge and/or attitudes. Due to the limited evidence base of peer education interventions, this review is broad in scope and will cover global peer education interventions covering all health areas. Although some peer education interventions are targeted towards specific populations, this review focuses on universal interventions available to an entire cohort of students (for example whole class or whole year group). The review aims to summarise the effectiveness of existing peer-led health interventions in schools. This is a review of quantitative data; the qualitative peer education literature will be published in a separate review.

## Methods

We followed the PICO (Population, Intervention, Comparator and Outcome) format to develop our research question. We completed the systematic review in accordance with the 2009 PRISMA statement [29] and registered it with PROSPERO (CRD42021229192).

### Search strategy and selection criteria

Five electronic databases were searched for eligible studies: CINAHL, Embase, ERIC, MEDLINE and PsycINFO. The list of search terms (see Supplementary Materials) were developed after scanning relevant literature for key terms. Searches took place during October 2020.

Once the search terms had been agreed amongst the study team, pilot searches were run to check that key texts were appearing. Search terms were subsequently refined and this process was repeated until all key texts appeared. Search strategies such as truncations were used to maximise results. No restrictions were placed on publication date, country or language.

### Inclusion/exclusion criteria

To be included studies had to be concerned with school-based peer education interventions designed to address aspects of the health of pupils aged 11–18 years old. We are interested in this age group in particular as it is a period when peers take on a particularly important role in young people’s lives. Peer education interventions concerned with health are defined here as interventions in which school-aged children deliver the education of other pupils for the purposes of improving health outcomes or awareness/literacy relating to health, including knowledge, behaviours and attitudes. Interventions must have taken place within a school, during school hours and must be universal, i.e. not targeted towards a specific sub-group of students or students with a particular health condition.

Where comparators/controls existed, they had to include non-exposure to the interventions concerned, exposure to a differing version of the same intervention, or exposure to the intervention within a substantially differing context.

Papers were excluded from data synthesis if they satisfied any of the following criteria:

1. Peer education interventions only concerned academic outcomes (e.g., reading and writing achievement).
2. Interventions concerning anger management, behavioural problems, or social skills.
3. Interventions concerning traffic safety, health and safety, avoidance of injuries, or first aid.
4. Interventions concerning cultural, social or political awareness (e.g., media literacy).
5. Interventions in which health outcomes are secondary to other outcomes (e.g., interventions focused on reading that indirectly improve self-esteem).
6. One-to-one mentoring interventions.
7. Conference abstracts, research briefings, commentaries, editorials, study protocol papers and pre-prints.

### Primary outcome(s)

1. Improvements in health, including health awareness and understanding as indicated by responses to questionnaires.
2. Reductions in health problems or risk-taking behaviours.

These outcomes may concern the peer educators and/or peer learners.

### Data extraction, selection and coding

Two reviewers independently screened all papers according to the inclusion criteria above using the Rayyan online review platform. In cases where the reviewers were uncertain, or where the decision was disputed, the decision was discussed and agreed among the wider research team. Two reviewers (SD and EW) then divided the papers between them and independently extracted the data, discussing and queries that arose with each other and the wider team.

Data extraction included the following:

1. Bibliographic details – authors, year of publication, nation in which intervention was carried out
2. Aims of the study
3. Description of study design
4. Sample size and demographic characteristics.
5. Context into which the intervention is introduced (characteristics of the school involved, the area in which the school is located, characterisations of the student body, relevant policy considerations).
6. Description of intervention (including duration of intervention).
7. Outcome measures (measurement tools, time points of data collection).
8. Data concerning improvements in health.

### Quality appraisal

We used the Mixed Methods Appraisal Tool (MMAT) to assess quality of reporting procedures. This tool consists of five specific quality rating items depending on study design (qualitative, quantitative randomized, quantitative non-randomized, quantitative descriptive and quantitative mixed methods). There are 5 quality questions specific to each study design, so all papers are rated between 0 to 5. The following ratings were used to summarise study quality; 0–1 indicating poor quality, 2–3 indicating average quality and 4–5 indicating high quality. Two reviewers (SD and EW) completed quality ratings on each paper and discussed any discrepancies between them.

Examples of randomized design quality questions included items such as: “*Is randomization appropriately performed*? And “*Are the groups comparable at baseline*?” Examples of non-randomized design quality questions included items such as: “*Are the participants representative of the target population?” and “Are there complete outcome data?”*

### Effectiveness summary

EW and SD completed data synthesis. Due to the volume of studies, and the large number and heterogeneity of outcome measures, in order to summarise effectiveness, we created the following scoring system to indicate effectiveness:

Significant effects are effects where there was an improvement in health-related outcomes either after the peer education intervention, or when compared to a control group, with a p value of <0.05. Due to the volume of studies and varied follow-up periods, we looked at effectiveness at first follow-up, which in the majority of papers was immediately post-intervention.

## Results

**Fig. 1:**
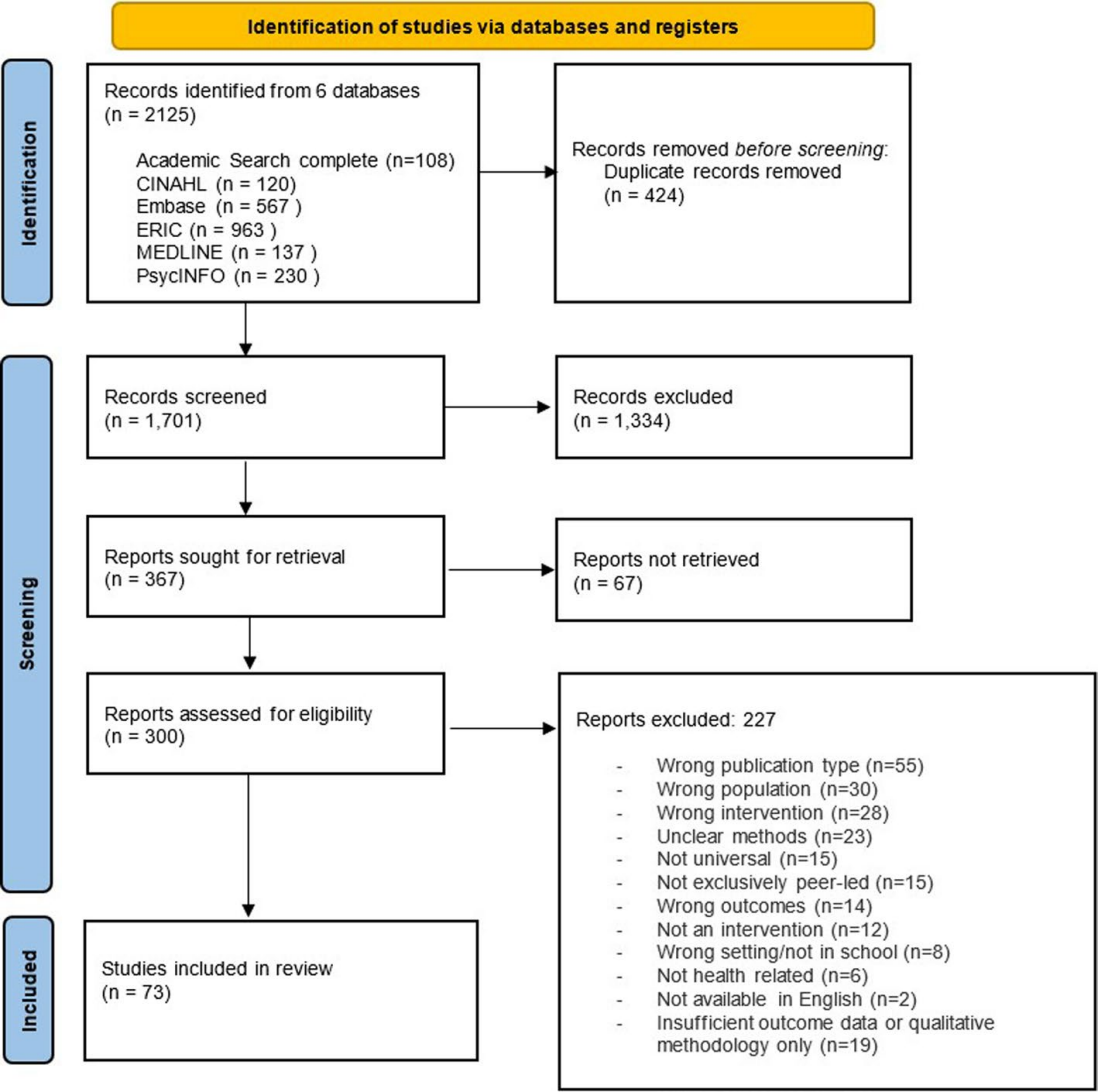
Prisma flow diagram of included studies

A total of 2125 articles were identified after the initial search and 73 articles were eligible for inclusion (see Fig. 1 for a flow diagram of the search). Study designs of the 73 articles were as follows: 23 were controlled trial designs (15 cluster or group randomised, 6 randomised controlled and 2 non-randomised). 15 used randomisation methods but were not controlled trials and the remaining 35 studies used uncontrolled non-randomised methods comparing intervention with a comparison group or using a pre-post survey.

### Health and geographical areas

**Fig. 2:**
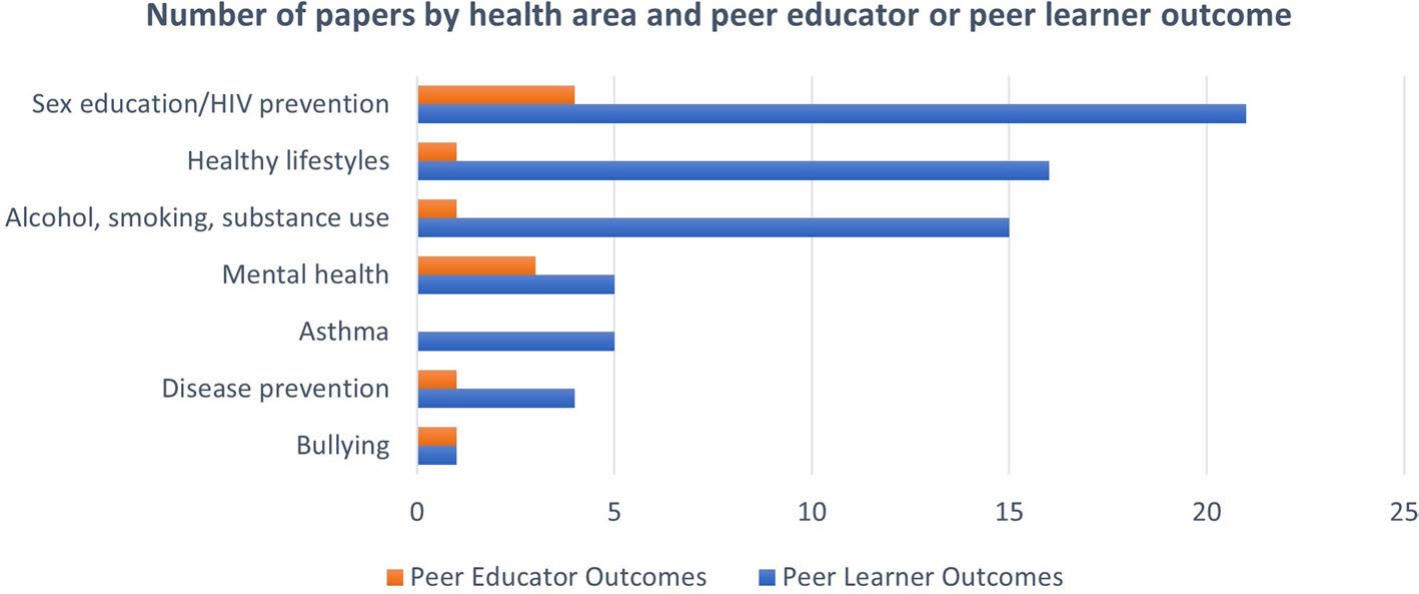
Number of papers by health area. NB See Supplementary Materials for full description of study designs and outcomes

The 73 quantitative papers included in this review demonstrated a wide range of health areas. The majority of papers evaluated interventions aimed at sex education/HIV prevention (*n* = 23), promoting healthy lifestyles (*n* = 17) and reducing alcohol, smoking and substance use (*n* = 16). Fig. 2 illustrates number of papers per health area by peer learner or peer educator outcome focus and Table 2 illustrates a summary of proportion of health areas, overall effectiveness and quality ratings.

Papers mainly focussed on peer learner outcomes (67/73, 91.8%), with only six papers (8.2%) focussing solely on peer educator outcomes and only five papers (6.8%) reporting on both peer learner and peer educator outcomes. The majority of papers that focussed on peer educator outcomes were those concerned with sex education (n = 4) and mental health (n = 3).

Papers typically reported knowledge, attitude and/or behavioural outcomes. Of the 73 papers, 42/73 (57.5%) reported knowledge outcomes, 43/73 (58.9%) reported attitude outcomes, 35/73 (47.9%) reported behavioural outcomes and 13/73 (17.8%) reported behavioural intentions.

**Fig. 3:**
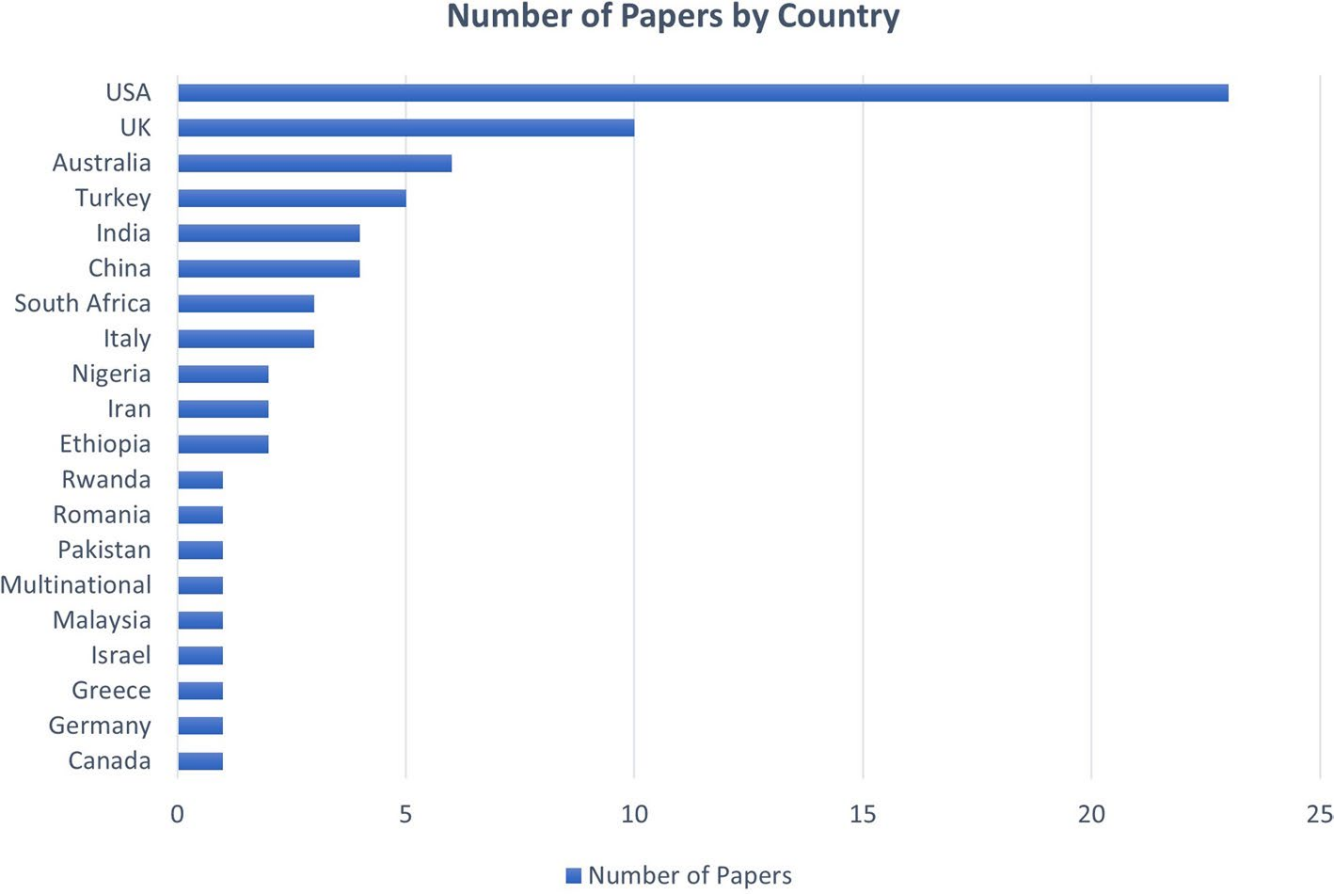
Summary of number of papers by country

As well as a broad range of health areas, the papers included in the review also spanned several different countries (Fig. 3).

We have summarised the results first by student type and then by health area.

### Results by student type

#### Summary of peer learner outcomes

Of the 67 papers reporting peer learner health outcomes, 35/67 (52.2%) showed evidence of effectiveness (as per our thresholds shown in Table 1), 8/67 (11.9%) showed mixed findings and 24/67 (35.8%) found limited or no evidence of effectiveness.

**Table 1:** Scoring thresholds for effectiveness

≥60% outcomes with significant effects	Suggests effectiveness
41–59% outcomes with significant effects	Mixed effectiveness
≤40% outcomes with significant effects	Suggests ineffectiveness

**Table 2:** Number of included papers by health area

Health area	Number of papers (% of total included)	Total participant number	Overall effectiveness rating^a^	Overall quality appraisal rating^b^
Sex education/HIV prevention	23 (31.5%)	51,354	Suggests ineffectiveness	Medium
Healthy lifestyles (exercise, nutrition, oral health, health information)	17 (23.3%)	21,172	Mixed effectiveness	High
Alcohol, smoking, substance use	16 (21.9%)	32,488	Mixed effectiveness	Medium
Mental health (incl. suicide prevention)	6 (8.2%)	6712	Suggests effectiveness	Medium
Asthma	5 6.8%)	2136	Suggests effectiveness	Medium
Disease prevention (including HIV, cervical cancer and hepatitis)	4 (5.4%)	8467	Suggests effectiveness	Medium
Bullying	2 (2.7%)	1607	Mixed effectiveness	High

^a^The proportion of ‘effective’ studies was calculated for each health area and then assigned an overall effectiveness rating based on our scoring thresholds in Table 1 (e.g. if ≥ 60% studies in a health area demonstrated effectiveness this health area was given an overall rating of effective)

^b^The average quality rating was calculated across all papers in health area, 0–1 indicating poor quality, 2–3 indicating average quality and 4–5 indicating high quality

Of the 35 papers that demonstrated effectiveness, 9/35 studies (25.7%) were rated as high quality. Therefore only 9/67 (13.4%) of the total papers showed evidence of effectiveness and were rated as high quality.

Twenty-one papers (31.3%) reported controlled trial designs (including 14 cluster or group randomised, and 5 randomised controlled and 2 non-randomised). Thirteen papers used randomisation methods but were not controlled trials and the remaining 33 papers used uncontrolled non-randomised methods comparing intervention with a comparison group or using a pre-post survey design.

#### Summary of peer educator outcomes

Of the 11 papers reporting on peer educator health outcomes, 4/11 (36.4%) showed evidence of effectiveness, 2/11 (18.1%) showed mixed findings and 5/11 (45.5%) showed limited or no evidence of effectiveness. Of the 4 papers showing evidence for effectiveness, 2 studies (50%) were rated as high quality.

Four papers had a randomised design comparing intervention vs. control or ‘peer educators vs. classmates’ one of which was a cluster randomised controlled trial. The remaining 7 papers used non-randomised intervention vs. control (*n* = 2) or pre-post survey designs (*n* = 5).

A full table of included studies, outcomes and effectiveness and quality ratings can be found in Supplementary Material 1.

### Results by health area

#### Sex education/HIV prevention

Twenty-three studies concerned sex education/HIV prevention [30–52]. 9/23 studies had a randomised design with the 8 studies comparing peer-led to teacher-led or ‘lessons as usual’ and one study comparing peer-led with nurse-led. 14/23 involved non-randomised designs comparing intervention vs. control or a pre-post survey design. Studies covered a wide geographical range, among which there were 7 US studies, but also studies from Canada, UK, Africa, South Africa, Turkey and Greece.

Of the twenty-three papers, 21 reported peer learner outcomes, 4 papers reported peer educator outcomes, with 2 papers reporting on both peer educator and peer learner outcomes. The mean number of participants across the studies was 2033 (range: *n* = 106–9000).

8/23 (34.8%) of studies showed evidence of effectiveness, and all studies demonstrating effectiveness consisted of knowledge and attitude outcomes rather than behavioural change.

Only 4/23 studies were rated high in quality (two of which showed evidence of effectiveness), whilst the majority of studies were rated medium quality (15/23) and 4/23 rated as low quality.

#### Healthy lifestyles (exercise, nutrition, oral health, health information)

Seventeen studies reported interventions addressing healthy lifestyles [53–69]. Of these papers, ten used a randomised controlled trial design primarily comparing peer-led vs. teacher-led or ‘lessons as usual’, but two oral health papers also used a dentist-led condition. Seven papers used non-randomised research designs comparing intervention vs. control or a pre-post survey design.

The most common focus was nutrition and exercise, but interventions also covered oral health, accessing health information online and interventions taking a more general approach to health improvement. Regarding geographical spread, 5/17 papers reported interventions carried out in the USA, with Australia, China, India and UK represented by two papers per country.

Sixteen of the seventeen papers reported peer learner outcomes, and only one reported peer educator outcomes. The mean number of participants per intervention was 1245 (range: *n* = 76–4576).

7/17 papers in this health area were shown to be effective, 8/17 were found to be ineffective, and 2/17 showed mixed results. In other words, less than half (41.1%) showed evidence of effectiveness. Of the studies demonstrating effectiveness, the outcomes largely centred around knowledge and attitudes, but one study did demonstrate positive behaviour change [62].

Over half of the studies (9/17) were rated as high quality, 4/17 were rated medium quality and 4/17 low quality. Of the studies showing evidence for effectiveness, 4/7 (57.1%) were rated as high quality.

#### Alcohol, smoking, substance use

Sixteen papers were classified within the category of alcohol, smoking and substance use [70–85]. Ten of these papers had a randomised design (including 3 cluster randomised controlled trials) comparing peer-led (intervention) vs. teacher-led (control). Six papers were non-randomised and used either a pre-post survey design or intervention vs. control. The 16 papers varied in quality with six rated ‘high quality’, seven rated ‘medium quality’, and three rated ‘low quality’. Studies took place across more than 10 countries with one study being conducted internationally. The mean number of participants across all studies was 2165 (range: n = 105–10,730).

Fifteen papers evaluated the effect of the intervention on peer learner outcomes and only one paper evaluated the effect of the intervention on peer educator outcomes. 8/16 (50%) papers showed evidence of effectiveness. 2/16 (12.5%) papers showed mixed findings and 6/16 (37.5%) showed little to no evidence for effectiveness, including the peer educator outcome paper. Of the eight papers demonstrating evidence for effectiveness, only four (50%) were rated as high quality.

Of the studies demonstrating effectiveness, there was a combination of knowledge, attitude and behavioural outcomes, but more evidence for positive changes in knowledge and attitude.

#### Mental health and well-being

Six studies assessed mental health and well-being [27, 86–90]. This category was inclusive of common mental health problems, self-harm and suicide prevention as well as broader topics such as self-esteem and social connectedness. Four of the six studies used non-randomised pre-post survey designs and two studies used randomised design, one of which was a cluster randomised controlled trial.

Of the six studies, 5/6 explored peer learner outcomes, 3/6 explored peer educator outcomes, 2 of which explored both peer learner and peer educator outcomes. The average sample size across the seven mental health studies was 1118 (range: *n* = 50–4128).

Study quality was mixed, with two studies rated as high quality, three medium quality and one low quality. Outcome measures largely consisted of knowledge and attitude questionnaires, help-seeking behaviour and help-seeking confidence as well as condition-specific measures including body satisfaction and self-report of emotional and behavioural difficulties.

The majority of mental health studies (5/6) were rated as showing evidence for effectiveness and one study was rated ineffective. Of the studies demonstrating effectiveness, only one reported positive behaviour change (help-seeking behaviours) and this behaviour changed was observed in peer educators as opposed to peer learners [86].

#### Disease prevention

Four studies assessed outcomes relating to disease prevention [91–94] which included hepatitis, tuberculosis, cervical cancer and blood borne diseases. All four studies focused on peer learner outcomes and one study also included peer educator outcomes. Three of the four studies were non-randomised pre-post survey designs and one study was randomised. The average sample size across the four studies was 2116 (range: 1265–2930).

Three out of the four studies (75%) showed evidence for effectiveness and one study showed mixed results. No studies were rated as high quality, three were rated medium and one was rated low.

Outcomes were largely knowledge or intention based. Studies showing effectiveness mostly related to knowledge, intentions and attitudes and one study did find a positive change in behaviour [93].

#### Asthma

Five included studies assessed asthma interventions [95–99]. 4/5 of these were randomised trials and one study used a non-randomised pre-post survey design. Average sample size across all studies was 427 (range: *n* = 203–935). Three studies took place in Australia and two in the US. All papers evaluated the impact of the intervention on peer learner outcomes with none focussing on peer educator outcomes.

4/5 studies showed evidence for effectiveness with only one study showing no evidence for effectiveness. All studies were rated as medium quality. Measures ranged from asthma knowledge, quality of life, school absenteeism, asthma attacks at school and asthma tests. Effectiveness was largely observed for knowledge outcomes, there was less evidence for asthma attacks or symptoms.

#### Bullying

Two studies conducted in Italy assessed bullying by evaluating the ‘NoTrap!’ anti-bullying intervention [100, 101]. The first study rated as high quality, evaluated two independent trials and focussed on peer learner outcomes (*n* = 622; *n* = 461). This study found significant reductions in victimization, bullying, cybervictimization and cyberbullying and was rated as high quality. The second study, rated as medium quality, focussed on peer educator outcomes (*n* = 524) and used a non-randomised, pre-post survey design but overall, only showed some evidence of effectiveness amongst males in terms of reduced victimization and increased prosocial behaviour and social support. No evidence was found for effectiveness among females.

## Discussion

Peer education interventions to improve student health cover a wide variety of topics and are used globally. This review aimed to summarise the results from peer education health interventions in secondary school students (aged 11–18-years-old), which were universal (rather than targeted interventions of sub-groups of students) and carried out at school.

Due to the heterogeneity of findings, range of health areas, types of studies and diversity of outcome measurements used, it was not possible to perform a meta-analysis or formal data synthesis to assess effectiveness. However, some broad conclusions can be made. A number of interventions appear to demonstrate evidence for effectiveness which indicates that peer education interventions can be an important school-based intervention for health improvement. Asthma interventions appeared to be particularly effective. In terms of outcome measures, the strongest evidence was for a positive change in knowledge and attitude measures, but there was less evidence overall for health behaviour outcomes which supports previous findings [20, 22].

Although many studies did demonstrate positive results, findings overall were very mixed and several studies were of poor quality. In addition to the shortcomings picked up on by our quality appraisal, many papers lacked methodological detail and clarity regarding the intervention procedure, particularly in regard to how peer educators were selected and trained, which seems to be an important factor in those studies that found positive results and was also emphasised in a previous review [10]. Further, there were widespread problems of data reporting including noting ‘significant’ results without providing any measure of effect size or between-study variability. Other problems included selective reporting of results, such as selective emphasis on anomalous positive results, or only revealing measures of statistical significance in the case of positive effects. Interestingly, there did not appear to be a relationship between study quality and findings, given that several studies rated as effective were rated both high and low quality with a similar picture for studies showing mixed effectiveness and ineffectiveness.

In terms of frequency of health areas covered, our findings are similar to a recent ‘review of reviews’ of peer education for health and wellbeing which found that the majority of reviews focused on sexual health and HIV/AIDS interventions [13]. This previous review focused on both children and adults, however, in line with our findings, it found mixed effectiveness and considerable diversity in methods, findings and rigour of evaluation. It was particularly noted that details of peer educator training were rarely provided in HIV/AIDS interventions which supports our findings. Notably, however, the quality of studies was actually highest for peer education programs in HIV/AIDS, which differed to our review which found few studies rated as high quality. This discrepancy may be due to the different measures used to assess quality. Like our study, this review concluded that each health area showed some promising results, but also pointed to a need for higher levels of quality and rigour in future evaluations.

Despite the rising prevalence in mental health difficulties, there were relatively few studies focused on mental health outcomes, particularly more general preventative approaches to mental health and well-being, with many of the included studies focusing on suicide prevention, self-harm or specific disorders. However, many of mental health studies included in this review showed evidence for effectiveness, suggesting peer education approaches for mental health should be further studied and evaluated.

Another key finding of our review is that papers tended to focus more on peer learner outcomes and therefore impacts of peer-led interventions on peer educators themselves appear to be under-explored. This has been reported by previous reviews [10] and highlights the importance of examining and comparing both peer educators’ and learners’ outcomes within studies. In this context, we found more evidence of peer learners benefitting from the interventions, with 55.2% of studies showing a positive effect, versus only 36.4% for peer educators. This contrasted with a previous review of mental health interventions that concluded peer educators seemed to yield more benefits from participating in the interventions, possibly due to the attention they are given during training and throughout the programmes [10].

Although common measures existed across studies, including health knowledge, health intentions, and health behaviours, many studies used novel or unvalidated measurements, indicating a need for more standardised health literacy measures and a need for future validation work in this area. This supports two systematic reviews carried out in 2015, firstly a review of health literacy measures which found a lack of comprehensive instruments to measure health literacy and suggested the need for the development of new instruments [102], and secondly a review of mental health literacy measures which found a number of unvalidated measures and lack of measures that measured all components of mental health literacy concurrently [103].

Although there are a number of existing reviews summarising the extent to which peer education may improve young peoples health, the literature is still lacking on why peer education is effective within the quantitative literature. It remains unclear which mechanisms involved in peer education lead to its effectiveness (or ineffectiveness). Although many peer education studies are grounded in theory such as Diffusion of Innovation Theory [104] and Bandura’s Social Cognitive/Social Learning Theory [105, 106], the literature is lacking a more nuanced analysis of the mechanisms through which peer education improve young people’s health. This is therefore a key area for future research.

A recent review of peer education and peer counselling for health and well-being highlights how peer education interventions are inherently difficult to quality control and evaluate [13], partly due to what makes peer education attractive; peer education defies the conventions of traditional formal education and allows young people to learn by more unstructured means, in more ‘real world’ ways, benefiting from meaningful examples and conversations with their peers. Although there are an increasing number of well-designed peer education studies [13], new evaluation methods may be needed given the complexity and multi-component nature of peer-education approaches (i.e., training, more informal teaching approaches and informal diffusion of knowledge).

## Limitations

Despite our review being comprehensive, we acknowledge certain limitations. ‘Peer education’ is a complex and widely contested term and therefore how studies described their approach varied substantially. This may have meant some relevant studies were not picked up from our initial search. A previous review [10] also noted this potential limitation, with unclear and heterogeneous methods precluding meta-analysis. Therefore, a consensus on how to define ‘peer education’ and using standardised measures to assess effectiveness would facilitate more definitive synthesis of the evidence. Another potential limitation of our approach is that we only searched scientific databases, and therefore could have missed important evidence in the grey literature as we retrieved a relatively small number of initial records (*n* = 2125). Despite this, given the wide variety of study type, age range, health area and country reviewed, this suggests our search strategy was fairly robust, and yielded results that were representative of the breadth in the current literature base.

This review focussed on universal peer education interventions delivered within the secondary school setting during school hours. Further research could explore the effectiveness of varying forms of peer education including 1:1 mentoring, more targeted (not universal) interventions, as well as peer education interventions in other settings including youth clubs or community and local organisations.

Due to the breadth of this review, we did not conduct a detailed comparison between knowledge, attitude and behavioural outcomes, however the studies demonstrating effectiveness tended to show positive change on knowledge and attitude outcomes, but less evidence was seen for positive behavioural change. This is in line with previous reviews which have suggested that peer education better improves health knowledge but often does not lead to behavioural gains [13, 107]. To this vein, it remains unclear the differential impact on behavioural intention and actual performance of behaviour, and therefore we urge future researchers to measure outcomes relating to knowledge and attitude, intentions, and actual behaviour in order to synthesise the evidence in a more standardised way. Although the literature is heterogeneous, there is available data to conduct distinct analysis on different outcome measures (knowledge, attitude and behaviour) to create a more nuanced understanding of each health area.

Given the large number of studies and variation in outcome measures (behaviour, knowledge, attitude), this review focussed on findings at first follow-up (usually immediately after intervention) and therefore the effectiveness findings are not likely to represent longer-term effects of peer education interventions, which would require further research. In addition, due to the low number of optimally designed randomised-controlled trials identified, our review could not meaningfully compare results between randomised and non-randomised studies. However, as more high quality trials continue to be published in this growing area of research, a future review could be conducted that looks into the effect of randomisation on young people’s outcomes. Our results also focused on p-values rather than effect sizes due to the large variability in how and what studies measures, future researchers should aim to agree on more standardises ways of measuring outcomes to enable better synthesis.

## Conclusion

To conclude, school-based peer education interventions occur worldwide and span a number of health areas. A number of interventions appear to demonstrate evidence for effectiveness, suggesting peer education may be a promising strategy for health improvement in schools. However overall evidence for effectiveness and study quality are mixed. Improvement in health-related knowledge was most common with less evidence for positive health behaviour change. In order to synthesise the evidence and make more confident conclusions, it is imperative that more robust, high-quality evaluations of peer-led interventions are conducted and that studies follow reporting guidelines to describe their methods and results in sufficient detail so that meta-analyses can be conducted. In addition, further research is needed to develop understanding of the intervention mechanisms that lead to health improvement in peer education approaches as well as more focussed work on standardising and validating health literacy and behaviour measurement tools.

### Pre-registration

This review was pre-registered on PROSPERO: CRD42021229192. One deviation was made from the original protocol which was the use of a different quality appraisal tool. Initially we had planned to use the Canadian Effective Public Health Project Practice (EPHPP) Quality Assessment Tool for Quantitative Studies and the Critical Appraisals Skills Programme (CASP) checklist for qualitative studies. The authors instead used a combined mixed methods tool (the Mixed Methods Appraisal Tool; MMAT) for both quantitative and qualitative studies. This was due to the large volume and variation of studies which meant there were benefits to using a single brief quality check tool across all included studies, allowing us to standardise scores across study types. The qualitative studies will be discussed in a separate realist review on key mechanisms of peer education interventions.

## Supplementary Information

**Additional file 1.**

## Data Availability

All data generated or analysed during this study are included in this published article and its supplementary information files.

## References

[1] Fazel M, Hoagwood K, Stephan S, Ford T (2014). Mental health interventions in schools 1: Mental health interventions in schools in high-income countries. Lancet Psychiatry.

[2] McKeganey SP, Neil. (2000). The rise and rise of peer education approaches. Drugs.

[3] Houlston C, Smith PK, Jessel J (2009). Investigating the extent and use of peer support initiatives in English schools. Educ Psychol.

[4] Winterton CI, Dunk RD, Wiles JR (2020). Peer-led team learning for introductory biology: relationships between peer-leader relatability, perceived role model status, and the potential influences of these variables on student learning gains. Discipli Interdisciplin Sci Educ Res.

[5] Blakemore SJ, Robbins TW (2012). Decision-making in the adolescent brain. Nat Neurosci.

[6] Lam CB, McHale SM, Crouter AC (2014). Time with peers from middle childhood to late adolescence: developmental course and adjustment correlates. Child Dev.

[7] NHS Digital. Mental Health of Children and Young People in England, 2020: Wave 1 follow up to the 2017 Survey. England: Health and Social Care Information Centre; 2020. Available online: https://digital.nhs.uk/data-and-information/publications/statistical/mental-health-of-children-and-young-people-in-england/2020-wave-1-follow-up#. Accessed 01 Aug 2022.

[8] Johnson EC, Robbins BA, Loui M (2015). What do students experience as peer leaders of learning teams? What Do Students Experience as Peer Leaders of Learning Teams?.

[9] Morgan D, Robbins J, Tripp J (2004). Celebrating the Achievements of Sex and Relationship Peer Educators: The Development of an Assessment Process. Sex Educ.

[10] King T, Fazel M (2021). Examining the mental health outcomes of school-based peer-led interventions on young people: A scoping review of range and a systematic review of effectiveness. PLoS One.

[11] Boyle J, Mattern CO, Lassiter JW, Ritzler JA (2011). Peer 2 peer: Efficacy of a course-based peer education intervention to increase physical activity among college students. J Am Coll Heal.

[12] Tolli MV (2012). Effectiveness of peer education interventions for HIV prevention, adolescent pregnancy prevention and sexual health promotion for young people: a systematic review of European studies. Health Educ Res.

[13] Topping KJ (2022). Peer Education and Peer Counselling for Health and Well-Being: A Review of Reviews. Int J Environ Res Public Health.

[14] Dennison S (2010). Peer mentoring: Untapped potential. J Nurs Educ.

[15] Thalluri J, O'Flaherty JA, Shepherd PL (2014). Classmate peer-coaching:" A Study Buddy Support scheme". J Peer Learn.

[16] Boulton MJ (2005). School peer counselling for bullying services as a source of social support: a study with secondary school pupils. Bri J Guidance Counsel.

[17] Abdi F, Simbar M (2013). The peer education approach in adolescents-narrative review article. Iran J Public Health.

[18] Mahat G, Scoloveno MA (2018). Effectiveness of adolescent peer education programs on reducing HIV/STI risk: an integrated review. Res Theory Nurs Pract.

[19] MacArthur GJ, Harrison S, Caldwell DM, Hickman M, Campbell R (2016). Peer-led interventions to prevent tobacco, alcohol and/or drug use among young people aged 11–21 years: a systematic review and meta-analysis. Addiction..

[20] Siddiqui M, Kataria I, Watson K, Chandra-Mouli V (2020). A systematic review of the evidence on peer education programmes for promoting the sexual and reproductive health of young people in India. Sex Reprod Health Matters.

[21] Kim CR, Free C (2008). Recent evaluations of the peer-led approach in adolescent sexual health education: A systematic review. Perspect Sex Reprod Health.

[22] Maticka-Tyndale E, Barnett JP (2010). Peer-led interventions to reduce HIV risk of youth: a review. Eval Program Plann.

[23] Mellanby AR, Rees JB, Tripp JH (2000). Peer-led and adult-led school health education: a critical review of available comparative research. Health Educ Res.

[24] Harden A, Oakley A, Oliver S (2001). Peer-delivered health promotion for young people: a systematic review of different study designs. Health Educ J.

[25] Yogev A, Ronen R (1982). Cross-age tutoring: Effects on tutors’ attributes. J Educ Res.

[26] Song Y, Loewenstein G, Shi Y (2018). Heterogeneous effects of peer tutoring: Evidence from rural Chinese middle schools. Res Econ.

[27] Ellis LA, Marsh HW, Craven RG (2009). Addressing the challenges faced by early adolescents: a mixed-method evaluation of the benefits of peer support. Am J Community Psychol.

[28] Shah S, McCallum GB, Wilson C, Saunders J, Chang AB (2017). Feasibility of a peer-led asthma and smoking prevention program (ASPP) in australian schools with high indigenous youth. Respirology..

[29] Moher D, Shamseer L, Clarke M, Ghersi D, Liberati A, Petticrew M (2015). Preferred reporting items for systematic review and meta-analysis protocols (PRISMA-P) 2015 statement. Syst Rev.

[30] Parwej S, Kumar R, Walia I, Aggarwal AK (2005). Reproductive health education intervention trial. Indian J Pediatr.

[31] Rotz D, Goesling B, Manlove J, Welti K, Trenholm C (2018). Impacts of a School-Wide, Peer-Led Approach to Sexuality Education: A Matched Comparison Group Design. J Sch Health.

[32] Mellanby AR, Newcombe RG, Rees J, Tripp JH (2001). A comparative study of peer-led and adult-led school sex education. Health Educ Res.

[33] Timol F, Vawda MY, Bhana A, Moolman B, Makoae M, Swartz S (2016). Addressing adolescents' risk and protective factors related to risky behaviours: Findings from a school-based peer-education evaluation in the Western Cape. SAHARA J.

[34] Aten MJ, Siegel DM, Enaharo M, Auinger P (2002). Keeping middle school students abstinent: outcomes of a primary prevention intervention. J Adolesc Health.

[35] Caron F, Godin G, Otis J, Lambert LD (2004). Evaluation of a theoretically based AIDS/STD peer education program on postponing sexual intercourse and on condom use among adolescents attending high school. Health Educ Res.

[36] Ebreo A, Feist-Price S, Siewe Y, Zimmerman RS (2002). Effects of peer education on the peer educators in a school-based HIV prevention program: where should peer education research go from here?...including commentary by Main DS. Health Educ Behav.

[37] Mason-Jones AJ, Flisher AJ, Mathews C (2011). Who are the peer educators? HIV prevention in South African schools. Health Educ Res.

[38] Menna T, Ali A, Worku A (2015). Effects of peer education intervention on HIV/AIDS related sexual behaviors of secondary school students in Addis Ababa, Ethiopia: a quasi-experimental study. Reprod Health.

[39] Siegel DM, Aten MJ, Roghmann KJ, Enaharo M (1998). Early effects of a school-based human immunodeficiency virus infection and sexual risk prevention intervention. Arch Pediatr Adolesc Med.

[40] Siegel DM, Aten MJ, Enaharo M (2001). Long-term effects of a middle school- and high school-based human immunodeficiency virus sexual risk prevention intervention. Arch Pediatr Adolesc Med..

[41] Stephenson JM, Strange V, Forrest S, Oakley A, Copas A, Allen E (2004). Pupil-led sex education in England (RIPPLE study): cluster-randomised intervention trial. Lancet..

[42] Stephenson J, Strange V, Allen E, Copas A, Johnson A, Bonell C (2008). The long-term effects of a peer-led sex education programme (RIPPLE): a cluster randomised trial in schools in England. PLoS Med.

[43] Strange V, Forrest S, Oakley A (2002). What influences peer-led sex education in the classroom? A view from the peer educators. Health Educ Res.

[44] Borgia P, Marinacci C, Schifano P, Perucci CA (2005). Is peer education the best approach for HIV prevention in schools? Findings from a randomized controlled trial. J Adolesc Health.

[45] Fisher JD, Fisher WA, Bryan AD, Misovich SJ (2002). Information-motivation-behavioral skills model-based HIV risk behavior change intervention for inner-city high school youth. Health Psychol.

[46] Huang H, Ye X, Cai Y, Shen L, Xu G, Shi R (2008). Study on peer-led school-based HIV/AIDS prevention among youths in a medium-sized city in China. Int J STD AIDS.

[47] Mahat G, Scoloveno MA (2010). HIV peer education: Relationships between adolescents' HIV/AIDS knowledge and self-efficacy. J HIV AIDS Soc Serv.

[48] Merakou K, Kourea-Kremastinou J (2006). Peer education in HIV prevention: an evaluation in schools. Eur J Pub Health.

[49] Michielsen K, Beauclair R, Delva W, Roelens K, Van Rossem R, Temmerman M (2012). Effectiveness of a peer-led HIV prevention intervention in secondary schools in Rwanda: results from a non-randomized controlled trial. BMC Public Health.

[50] Ozcebe H, Akin L, Aslan D (2004). A peer education example on HIV/AIDS at a high school in Ankara. Turk J Pediatr.

[51] Visser MJ (2007). HIV/AIDS prevention through peer education and support in secondary schools in South Africa. Sahara J.

[52] Jennings JM, Howard S, Perotte CL (2014). Effects of a school-based sexuality education program on peer educators: the Teen PEP model. Health Educ Res.

[53] Cohen RY, Felix MR, Brownell KD (1989). The role of parents and older peers in school-based cardiovascular prevention programs: implications for program development. Health Educ Q.

[54] Lytle LA, Murray DM, Perry CL, Story M, Birnbaum AS, Kubik MY (2004). School-based approaches to affect adolescents' diets: results from the TEENS study. Health Educ Behav.

[55] Shankar P, Sievers D, Sharma R (2020). Evaluating the Impact of a School-Based Youth-Led Health Education Program for Adolescent Females in Mumbai, India. Ann Glob Health.

[56] Forneris T, Fries E, Meyer A, Buzzard M, Uguy S, Ramakrishnan R (2010). Results of a rural school-based peer-led intervention for youth: goals for health. J Sch Health..

[57] Foley BC, Shrewsbury VA, Hardy LL, Flood VM, Byth K, Shah S (2017). Evaluation of a peer education program on student leaders' energy balance-related behaviors. BMC Public Health.

[58] Cui Z, Shah S, Yan L, Pan Y, Gao A, Shi X (2012). Effect of a school-based peer education intervention on physical activity and sedentary behaviour in Chinese adolescents: a pilot study. BMJ Open.

[59] Ishak SIZS, Siew CY, Mohd Shariff Z, Mun CY, Moh TN (2019). Effectiveness of a school-based, peer-led intervention program on the adolescents' body composition, eating behaviors and health-related quality of life. Ann Nutr Metab.

[60] Tamiru D, Argaw A, Gerbaba M, Ayana G, Nigussie A, Jisha H (2017). Enhancing Personal Hygiene Behavior and Competency of Elementary School Adolescents through Peer-Led Approach and School-Friendly: A Quasi-Experimental Study. Ethiop J Health Sci.

[61] Bogart LM, Elliott MN, Cowgill BO, Klein DJ, Hawes-Dawson J, Uyeda K (2016). Two-Year BMI Outcomes From a School-Based Intervention for Nutrition and Exercise: A Randomized Trial. Pediatrics..

[62] Shrewsbury VA, Venchiarutti RL, Hardy LL, Foley BC, Bonnefin A, Byth K (2020). Impact and cost of the peer-led Students As LifeStyle Activists programme in high schools. Health Educ J.

[63] Bell SL, Audrey S, Cooper AR, Noble S, Campbell R (2017). Lessons from a peer-led obesity prevention programme in English schools. Health Promot Int.

[64] Bogart LM, Cowgill BO, Elliott MN, Klein DJ, Hawes-Dawson J, Uyeda K (2014). A randomized controlled trial of students for nutrition and eXercise: a community-based participatory research study. J Adolesc Health.

[65] Ajuwon GA, Ajuwon AJ (2019). Teaching high school students to use online consumer health resources on mobile phones: outcome of a pilot project in Oyo State, Nigeria. J Med Lib Assoc.

[66] Haleem A, Siddiqui MI, Khan AA (2012). School-based strategies for oral health education of adolescents--a cluster randomized controlled trial. BMC Oral Health.

[67] Vangipuram S, Jha A, Raju R, Bashyam M (2016). Effectiveness of peer group and conventional method (Dentist) of oral health education programme among 12-15 year old school children - A randomized controlled trial. J Clin Diagn Res.

[68] Sebire SJ, Jago R, Banfield K, Edwards MJ, Campbell R, Kipping R (2018). Results of a feasibility cluster randomised controlled trial of a peer-led school-based intervention to increase the physical activity of adolescent girls (PLAN-A). Int J Behav Nutr Phys Act.

[69] Ping HU, Lingli HAN, Manoj S, Huan Z, Yong Z, Hui LI (2014). Evaluation of Cognitive and Behavioral Effects of Peer Education Model-Based Intervention to Sun Safe in Children. Iran J Public Health.

[70] Perry CL, Grant M, Ernberg G, Florenzano RU, Langdon MC, Myeni AD (1989). WHO Collaborative Study on Alcohol Education and Young People: outcomes of a four-country pilot study. Int J Addict.

[71] Weichold K, Silbereisen RK (2012). Peers and teachers as facilitators of the life skills program IPSY - Results from a pilot study. Sucht..

[72] Lachausse RG (2008). The effectiveness of a multimedia program to prevent fetal alcohol syndrome. Health Promot Pract.

[73] Erhard R (1999). Peer-led and adult-led programs--student perceptions. J Drug Educ.

[74] Audrey S, Holliday J, Campbell R (2006). It's good to talk: adolescent perspectives of an informal, peer-led intervention to reduce smoking. Soc Sci Med.

[75] Bloor M, Frankland J, Langdon NP, Robinson M, Allerston S, Catherine A (1999). A controlled evaluation of an intensive, peer-led, schools-based, anti-smoking programme. Health Educ J.

[76] Campbell R, Starkey F, Holliday J, Audrey S, Bloor M, Parry-Langdon N (2008). An informal school-based peer-led intervention for smoking prevention in adolescence (ASSIST): a cluster randomised trial. Lancet..

[77] Lotrean LM, Dijk F, Mesters I, Ionut C, De Vries H (2010). Evaluation of a peer-led smoking prevention programme for Romanian adolescents. Health Educ Res.

[78] Mall ASK, Bhagyalaxmi A (2017). An Informal School-based, Peer-led Intervention for Prevention of Tobacco Consumption in Adolescence: A Cluster Randomized Trial in Rural Gandhinagar. Indian J Community Med.

[79] Mohammadi M, Ghaleiha A, Rahnama R (2019). Effectiveness of a peer-led behavioral intervention program on tobacco use-related knowledge, attitude, normative beliefs, and intention to smoke among adolescents at Iranian Public High Schools. Int J Prev Med.

[80] Murray DM, Richards PS, Luepker RV, Johnson CA (1987). The prevention of cigarette smoking in children: two- and three-year follow-up comparisons of four prevention strategies. J Behav Med.

[81] Perry CL (1980). Peer Teaching and Smoking Prevention among Junior High Students. Adolescence..

[82] Botvin GJ, Baker E, Filazzola AD, Botvin EM (1990). A cognitive-behavioral approach to substance abuse prevention: one-year follow-up. Addict Behav.

[83] Demirezen D, Karaca A, Konuk Sener D, Ankarali H (2019). Agents of change: the role of the peer education program in preventing adolescent substance abuse. J Child Adolesc Subst Abuse.

[84] Severson HH, Glasgow R, Wirt R, Brozovsky P, Zoref L, Black C (1991). Preventing the use of smokeless tobacco and cigarettes by teens: results of a classroom intervention. Health Educ Res.

[85] Aslan D, Sahin A (2007). Adolescent peers and anti-smoking activities. Promot Educ.

[86] Wyman PA, Brown CH, LoMurray M, Schmeelk-Cone K, Petrova M, Yu Q (2010). An outcome evaluation of the Sources of Strength suicide prevention program delivered by adolescent peer leaders in high schools. Am J Public Health.

[87] Ciao AC, Latner JD, Brown KE, Ebneter DS, Becker CB (2015). Effectiveness of a peer-delivered dissonance-based program in reducing eating disorder risk factors in high school girls. Int J Eat Disord.

[88] Eisenstein C, Zamperoni V, Humphrey N, Deighton J, Wolpert M, Rosan C (2019). Evaluating the peer education project in secondary schools. J Public Ment Health..

[89] Kaveh MH, Hesampour M, Ghahremani L, Tabatabaee HR (2014). The effects of a peer-led training program on female students' self-esteem in public secondary schools in Shiraz. J Adv Med Educ Prof.

[90] Parikh SV, Taubman DS, Antoun C, Cranford J, Foster CE, Grambeau M (2018). The Michigan Peer-to-Peer Depression Awareness Program: School-Based Prevention to Address Depression Among Teens. Psychiatr Serv.

[91] Isik M, Set T, Khan AS, Avsar UZ, Cansever Z, Acemoglu H (2013). Prevalence of Blood Brotherhood among High School Students in Erzurum and the Effect of Peer-led Education on this Practice. Eurasian J Med.

[92] Sadoh AE, Okonkwo C, Nwaneri DU, Ogboghodo BC, Eregie C, Oviawe O (2018). Effect of peer education on knowledge of human papilloma virus and cervical cancer among female adolescent students in Benin city, Nigeria. Ann Global Health.

[93] Acemoglu H, Palanci Y, Set T, Vancelik S, Isik M, Polat H (2011). An intervention study for viral hepatitis: Peer-led health education among high school students. Saudi Med J.

[94] Liu Q, Liu L, Vu H, Liu X, Tang S, Wang H (2015). Comparison between peer-led and teacher-led education in tuberculosis prevention in rural middle schools in Chongqing, China. Asia Pac J Public Health.

[95] Al-sheyab N, Gallagher R, Crisp J, Shah S (2012). Peer-led education for adolescents with asthma in Jordan: a cluster-randomized controlled trial. Pediatrics..

[96] Al-sheyab NA, Alomari MA, Shah S, Gallagher R (2016). "Class smoke-free" pledge impacts on nicotine dependence in male adolescents: A cluster randomized controlled trial. J Subst Abus.

[97] Gibson PG, Shah S, Mamoon HA (1998). Peer-led asthma education for adolescents: impact evaluation. J Adolesc Health.

[98] McCallum GB, Chang AB, Wilson CA, Petsky HL, Saunders J, Pizzutto SJ (2017). Feasibility of a Peer-Led Asthma and Smoking Prevention Project in Australian Schools with High Indigenous Youth. Front Pediatr.

[99] Shah S, Peat JK, Mazurski EJ, Wang H, Sindhusake D, Bruce C (2001). Effect of peer led programme for asthma education in adolescents: cluster randomised controlled trial. BMJ..

[100] Palladino BE, Nocentini A, Menesini E (2016). Evidence-based intervention against bullying and cyberbullying: Evaluation of the NoTrap! program in two independent trials. Aggress Behav.

[101] Zambuto V, Palladino BE, Nocentini A, Menesini E (2019). Why do some students want to be actively involved as peer educators, while others do not? Findings from NoTrap! Anti-bullying and anti-cyberbullying program. Eur J Dev Psychol.

[102] Tavousi M, Ebadi M, Fattahi E, Jahangiry L, Hashemi A, Hashemiparast M (2015). Health literacy measures: A systematic review of the literature.

[103] Wei Y, McGrath PJ, Hayden J, Kutcher S (2015). Mental health literacy measures evaluating knowledge, attitudes and help-seeking: a scoping review. BMC Psychiatry.

[104] Kaminski J (2011). Diffusion of innovation theory. Can J Nurs Inform.

[105] Bandura A, Walters RH (1977). Social learning theory: Englewood cliffs Prentice Hall.

[106] Bandura A, Smith KG, Hitt MA (2019). The evolution of social cognitive theory. Great Minds in Management.

[107] Milburn K (1995). A critical review of peer education with young people with special reference to sexual health. Health Educ Res.

