# Supplementary material for: School-based peer education interventions to improve health: a global systematic review of effectiveness
Source: BMC Public Health. 2022 Dec 2;22:2247. doi: 10.1186/s12889-022-14688-3 (PMC9719233; doi:10.1186/s12889-022-14688-3)
Supplement: Supplementary file 1 — Additional file 1. [file 12889_2022_14688_MOESM1_ESM.pdf]

Supplementary Material 1: Full table of included study details, effectiveness and quality ratings for peer learner outcomes

Author, Year	Study Design	Nation	Sample size	Health Area	Outcome & effectiveness	Effectiveness	Quality Score	Quality Rating
Aslan & Sahin, 2007	Non-randomised, pre-post design	Turkey	279	Alcohol, smoking, substance use	↑<br>Knowledge & attitudes<br>(See paper for a breakdown of results as reported by the author at the level of multiple individual statements)	Effective	2	Medium
Bloor et al. 1999	Non-randomised, intervention vs. control	UK	1300	Alcohol, smoking, substance use	↔<br>Change in smoking behaviour immediately post intervention (p=0.22)<br>↑<br>Smoking abstinence in ex-smokers (p =0.004)	Mixed	1	Low
Botvin et al. 1990	Randomised, peer-led vs. teacher-led	USA	1185	Alcohol, smoking, substance use	Peer intervention, peer intervention + booster<br>↔,↑<br>Smoking use (NS), (<.01)<br>↔,↔<br>Alcohol use (NS), (NS)<br>↔,↑<br>Marijuana use (NS), (<.05)<br>↑,↑<br>Knowledge: Tobacco (p < .0001), (p < .0001)<br>↑,↑<br>Knowledge: Alcohol (p < .01), (p < .0001)<br>↔, ↔<br>Knowledge: Marijuana (NS), (NS)<br>↔,↑<br>Attitudes: Tobacco (NS), (p < .01)<br>↔, ↔<br>Attitudes: Alcohol (NS), (NS)<br>↔, ↔<br>Marijuana (NS), (NS)<br>↔,↔<br>Personality: Assertiveness (NS), (NS)	Ineffective	2	Medium

					⟷, ⟷<br>Personality: Locus of control (NS), (p < .01)<br>⟷, ↑<br>Personality: Social anxiety (NS), (NS)<br>⟷, ⟷<br>Personality: Self-esteem (NS), (NS)<br>⟷, ⟷<br>Personality: Smoking influenceability (NS), (NS)<br>⟷, ⟷<br>Personality: General influenceability (NS), (NS)			
Campbell et al. 2008	Cluster randomised controlled trial, intervention vs. control	UK	10,730	Alcohol, smoking, substance use	↑<br>All students: Smoking prevalence immediately after intervention (*p* =0.031)	Effective	5	High
Demirezen et al. 2020	Non-randomised, pre-post survey	Turkey	663	Alcohol, smoking, substance use	↑<br>Knowledge and attitude scores (*p* = < .01)<br>↑ ⟷<br>Incidence of smoking: just once (*p* =<.001), yes (NS)<br>⟷⟷<br>Incidence of alcohol use: just once (NS), yes (NS)<br>⟷⟷<br>Incidence of cannabis use: just once (NS), yes (NS)<br>⟷⟷<br>Incidence of using ecstasy: just once (NS), yes (NS)<br>⟷⟷<br>Incidence of using volatiles: just once (NS), yes (NS)<br>↑⟷<br>Incidence of using non-prescription drugs: just once (*p* =<.001), yes (NS)	Ineffective	3	Medium
Erhard, 1999	Non-randomised, peer-led vs. adult-led	Israel	2447	Alcohol, smoking, substance use	↑<br>Evaluation of knowledge: (*p* =< .05)<br>↑<br>Evaluation of motivation: (*p* =< .05)<br>↑	Effective	2	Medium

					Increased curiosity: (*p* =< .05)<br>↑<br>Facilitators as an address: (*p* =< .05)<br>↑<br>Improved relations: (*p* =< .05)<br>↑<br>Satisfaction: (*p* = < .05)			
LaChause, 2008	Randomised, intervention vs. control	USA	114	Alcohol, smoking, substance use	↑<br>FAS knowledge (*p* =.01)<br>↔<br>Attitudes toward alcohol use during pregnancy (NS)<br>↔<br>Likelihood to use alcohol during pregnancy (NS)<br>↔<br>Intention to use alcohol during pregnancy (NS)	Ineffective	1	Low
Lotrean et al. 2010	Randomised, intervention vs. control	Romania	1071	Alcohol, smoking, substance use	↑<br>Pros of smoking (*p* = <0.01)<br>↔<br>Cons of smoking (NS)<br>↑<br>Self-efficacy social (*p* = <0.05)<br>↔<br>Self-efficacy emotional (NS)<br>↔<br>Self-efficacy situational (NS)<br>↑<br>Intention to smoke (*p*=<0.05)	Mixed	4	High
Mall & Bhagyalaxmi, 2017	Cluster randomised trial, intervention vs. control	India	802	Alcohol, smoking, substance use	↑<br>Tobacco consumption at follow-up (*p* =< 0.01)	Effective	1	Low
Mohammadi et al. 2019	Cluster randomised trial,	Iran	1807	Alcohol, smoking, substance use	↑<br>Smoking-related knowledge: (*p*= <0.001)<br>↔<br>Attitude toward smoking : (*p*=0.124)	Effective	4	High

	intervention vs. control				↑<br>Normative beliefs: (*p*=<0.001)<br>↑<br>Intention to tobacco use: (*p* = <0.013)<br>↔			
Murray et al. 1987	Non-randomised, pre-post survey	USA	Study 1: 3154<br>Study 2: 3820	Alcohol, smoking, substance use	↑<br>Reduced smoking incidence at 1 year<br>↑<br>Smoking intensity levels at 1 year<br>↑<br>Peer-led social influence condition	Effective	2	Medium
Perry et al. 1980	Non-randomised, intervention vs. control	USA	707	Alcohol, smoking, substance use	↑<br>Smoked in past week<br>↑<br>Smoked in past month	Effective	4	High
Perry et al. 1989	Randomised, peer-led, teacher-led, control	Multinational (Australia, Chile, Norway Switzerland)	2536	Alcohol, smoking, substance use	↑<br>Alcohol use (*p* =<.005)<br>↑<br>Knowledge (*p* =<.005)<br>↑<br>Attitudes (*p* =<.005)<br>↓<br>Skills (*p* =<.08)<br>↑<br>Friends' Drinking (*p* =<.005)	Effective	2	Medium
Severson et al. 1991	Randomised, peer-led vs. teacher-led	USA	1768	Alcohol, smoking, substance use	↑<br>Smokeless tobacco use in males (*p* =<0.05)<br>↑<br>Smokeless tobacco cessation rate<br>↔<br>Cigarette Smoking (NS)<br>↓<br>Carbon monoxide level (*p* =<0.05)<br>↔<br>Alcohol use (NS)	Ineffective	4	High

					↔<br>Marijuana use (NS)			
Weichold & Silbereisen, 2012	Randomised, peer-led, teacher-led, control	Germany	105	Alcohol, smoking, substance use	↓<br>Alcohol consumption – higher in peer-led condition compared to teacher-led (*p* = < .05 for T2) than in control (*p* = < .01 for T2).<br><br>↓<br>Frequency of drinking - higher in peer-led condition compared to teacher-led condition (*p* = < .001 for T2)<br><br>↓<br>Cigarette consumption – lower in students of the teacher-led condition compared to peer-led condition (*p* = < .05 for T2 and to those of the control group (*p* =< .01 for T2).<br><br>↓<br>Resistance skills - lower in the peer-led intervention (*p* = < .05 for T2) compared to control group (*p* =< .05 for T2)	Ineffective	2	Medium
Al-Sheyab et al. 2012	Cluster randomised controlled trial, intervention vs. control	USA	240	Asthma	↑<br>Health-related quality of life (*p* =<.05)<br>↑<br>Self-efficacy to resist smoking (*p* =<.05)<br>↑<br>Knowledge of asthma self-management (*p* =<.05)	Effective	2	Medium
Al-Sheyab et al. 2016	Cluster, randomised controlled trial, intervention vs. control	USA	486	Asthma	↑<br>Smoking related knowledge (*p* = < 0.001)<br>↑<br>Level of nicotine dependence (*p* = < 0.001)<br>↑<br>Asthmas control scores (*p* =< 0.001)	Effective	2	Medium
Gibson et al. 1998	Controlled trial,	Australia	935	Asthma	↑<br>Mean scores of asthma Knowledge (*p* =< 0.001)	Effective	3	Medium

	intervention vs. control							
McCallum et al. 2017	Non-randomised, pre – post survey	Australia	203	Asthma	⟷ ‘Asthma control test score: no change	Ineffective	2	Medium
Shah et al. 2001	Cluster randomised controlled trial, intervention vs. control	Australia	272	Asthma	↑ Quality of life (*p* =0.01)<br>↑ Activities domain (*p* =0.028)<br>↑ Emotions domain (males only), (*p* =0.02)<br>↑ School 6bsenteeism (*p* =<.05)<br>⟷ Asthma attacks at school (NS)	Effective	3	Medium
Palladino et al. 2016	Non-randomised, intervention vs. control	Italy	Trial 1: 622<br>Trial 2: 461	Bullying	↑ Victimization: (*p* =.003)<br>↑ Bullying – (*p* =.010)<br>↑ Cybervictimization – (*p* =0.002)<br>↑ Cyberbullying – (*p* = 0.009)	Effective	4	High
Isik et al. 2013	Non-randomised, cross-sectional survey	Turkey	2829	Disease prevention	↑ Intention to participate in BB rituals (*p* =<0.001)	Effective	3	Medium
Sadoh et al. 2018	Non-randomised, pre-post survey	Nigeria	1443	Disease prevention	↑ Awareness of cervical cancer<br>↑ Knowledge of risk factors and cause of cervical cancer	Effective	2	Medium
Acemoglu, 2011	Non-randomised,	Turkey	2930	Disease prevention	↑ Knowledge scores (*p* =0.001)	Effective	1	Low

	pre-post survey				↑<br>Attitudes of students towards people infected with hepatitis virus (*p* =0.002)<br>↑<br>Change in behaviours (*p* =0.000)			
Liu et al. 2015	Randomised, peer-led vs. teacher-led	China	1265	Disease prevention	↑<br>Pre-post knowledge, attitude & practice<br>↓<br>Knowledge, attitude & practice (larger improvement in teacher-led, *p* =<.01)	Mixed	3	Medium
Ajuwon & Ajuwon, 2019	Non-randomised, pre-post survey	Nigeria	120	Healthy lifestyles	↑<br>Knowledge of CHI resources<br>↑<br>Understanding of roles as peer educators	Effective	2	Medium
Bell et al. 2017	Randomised, intervention vs. control	UK	928	Healthy lifestyles	↑<br>Consumption of breakfast (*p* =0.004)<br>⟷<br>Consumption of at least three portions of fruit a day (*p*= 0.055)<br>⟷<br>No statistically significant difference in the reported consumption of other target foods<br>↑<br>Counts per minute (CPM)<br>↑<br>Sedentary minutes per day<br>⟷<br>MVPA minutes per day	Mixed	4	High
Bogart et al. 2014	Randomised trial, intervention vs. wait-list control	USA	2997	Healthy lifestyles	↑<br>Fruit servings (*p* =<.10)<br>⟷<br>Vegetable servings: ns<br>↑<br>All lunches (*p* =<.01)<br>↑<br>Free/reduced lunch (*p* =<.001)	Effective	4	High

					↑<br>Full-price lunch (*p* =<.01)<br>↑<br>Cafeteria attitudes (*p* =< .05)<br>↑<br>Tap water attitudes (*p* =< .05)<br>↑<br>Knowledge about healthy eating/physical activity (*p* = < .05)<br>↑<br>Intentions to drink tap water (*p* = < .05)<br>↑<br>Intentions to drink from refillable bottled (*p* =< .05)<br>↑<br>Tap water consumption (*p* = < .05)<br>⟷<br>Refillable bottle use (NS)			
Bogart et al. 2016	Randomised trial, intervention vs. wait-list control	USA	4576	Healthy lifestyles	↑<br>Students obese at seventh grade (*p* =<.01)<br>⟷<br>Students overweight at seventh grade (no intervention effect)<br>⟷<br>Students healthy weight/ underweight at seventh grade (no intervention effect)	Ineffective	2	Medium
Cohen et al. 1989	Non-randomised, peer-led vs. teacher-led	USA	886	Healthy lifestyles	⟷<br>Blood pressure behavioural capabilities (NS)<br>⟷<br>Smoking experimentation (NS)<br>⟷<br>Nutrition behavioural capabilities (NS)<br>⟷<br>Family discussion (NS)	Ineffective	3	Medium
Cui et al. 2012	Randomised, intervention vs. control	China	682	Healthy lifestyles	⟷<br>Decrease in time spent on sedentary behaviour: 3 months (*p* = 0.21)<br>⟷	Ineffective	3	Medium

					Decrease in time spent on sedentary behaviour on weekdays: 3 months (*p* =0.21)<br>↔<br>Decrease in time spent on sedentary behaviour on weekends: 3 months (*p* =0.25)<br>↔,<br>Time spent on computer: 3 months: (*p* =0.13)<br>↔<br>Time spent on computer on weekdays: 3 months - (*p* =0.07)<br>↔<br>Time spent on computer on weekend: 3 months (*p* =0.27)<br>↔<br>Time spent on Television and DVD: 3 months (*p* = 0.13)<br>↔<br>Time spent on Video games: 3 months (*p*=0.26)<br>↔<br>Time spent on extracurricular reading, writing, drawing and listening to music: 3 months (*p* = 0.4)<br>↔<br>Time spent on passive commuting: 3 months (*p* =0.22)<br>↔<br>Time spent sitting and talking: 3 months (*p* =0.16)			
Forneris et al 2010	Randomised, intervention vs. wait-list control	USA	523	Healthy lifestyles	↑<br>Self-Efficacy to Eat Healthy (*p* =<.05)<br>↔<br>Perceived Taste of Low-Fat Foods “not significant”<br>↑<br>Fat and Fiber Knowledge (*p* =<.003)<br>↔<br>Fat Food Frequency Score (*p* = .98)<br>↔<br>Fiber Food Frequency Score (*p* = .89)	Ineffective	1	Low

					⟷<br>Fruit and Vegetable Food Frequency Score (*p* =.76)			
Haleem et al. 2012	Cluster randomised controlled trial, per-led, dentist-led and teacher-led	Pakistan	1657	Healthy lifestyles	⟷<br>Oral health knowledge (NS)<br>⟷<br>Oral hygiene status (NS)<br>↑<br>Oral health behaviour (*p* =<0.05)	Ineffective	5	High
Ishak et al. 2019	Non-randomised, intervention vs. control	Malaysia	76	Healthy lifestyles	↑<br>Knowledge score at time interval one (*p* = 0.006)<br>↑<br>Knowledge score at time interval two (*p* = 0.005)	Effective	4	High
Lytle et al. 2004	Group-randomised trial, intervention vs. control	USA	2883	Healthy lifestyles	⟷<br>Scores for total fruit and veg (*p* =.11)<br>⟷<br>Scores for energy from total fat (*p* =.90)<br>↑<br>Food choice score (*p* =p=.01)	Ineffective	4	High
Ping et al. 2014	Cluster randomised control trial, intervention vs. control	China	645	Healthy lifestyles	↑<br>Sun safe knowledge (*p* =<.001)	Effective	4	High
Sebire et al. 2018	Cluster randomised controlled trial, intervention vs. control	UK	427	Healthy lifestyles	⟷<br>Mean weekday moderate-to-vigorous physical activity<br>⟷<br>Mean weekend moderate-to-vigorous physical activity<br>↑<br>Mean weekday sedentary (mins)<br>⟷<br>Mean weekend sedentary (mins)	Ineffective	5	High
Shankar et al. 2020	Non-randomised,	India	755	Healthy lifestyles	↑<br>Health knowledge (*p* =< 0.001)	Effective	1	Low

	pre-post survey				↑<br>Health-related attitudes (*p* =< 0.001)			
Shrewsbury et al. 2020	Non-randomised, pre-post survey	Australia	2056	Healthy lifestyles	↔ ↑<br>Daily breakfast (behaviour, *p* =.675, intention, *p* =<.05)<br>↑ ↑<br>Fruit ⩾2 serves/day (behaviour, *p*=<.001), intention, *p*=<.001)<br>↔ ↑<br>Vegetables⩾5 serves/day (behaviour, *p*=.156, intention, *p*=<.01)<br>↑<br><1 cup/day SSB (behaviour, *p*=<001.)<br>↔ ↑<br>Screen-time ⩽2 hours/day (behaviour, *p*=<.01, intention, *p*=<.001)	Effective	5	High
Tamiru et al. 2017	Non-randomised, pre-post survey	Ethiopia	992	Healthy lifestyles	↔<br>Dietary diversity score (*p*=0.093)<br>↔<br>Food variety score (*p*=0.075<br>↑<br>Animal source food intake (*p*<0.001)	Ineffective	1	Low
Vangipuram et al. 2016	Randomised controlled trial, peer-led, dentist-led & control	India	450	Healthy lifestyles	↑<br>Oral health knowledge<br>↑<br>Attitude<br>↑<br>Oral hygiene practices<br>↑<br>Oral health status<br>↑<br>Oral health behaviour	Effective	1	Low
Ciao et al. 2015	Non-randomised, intervention	USA	50	Mental health	↑<br>Internalization (*p* =<0.001)<br>↑	Effective	3	Medium

	vs. wait-list control				Body dissatisfaction (*p* =<0.005)<br>↑<br>Eating pathology (*p* =<0.012)			
Eisenstein et al. 2019	Non-randomised, pre-post survey	UK	950	Mental health	↑<br>Understanding of key terms (*p* =<0.001)<br>↑<br>Key skills (*p* =<0.001)<br>↑<br>Confidence to talk about mental health (*p* =0.03)<br>⟷<br>Knowledge of information and resources (*p* = 0.07)<br>↑<br>Readiness to support others (*p* <0.001)<br>↑<br>Emotional difficulties (*p* = 0.005)<br>⟷<br>Behavioural difficulties (*p* = 0.10)<br>↑<br>School climate (*p* <0.001)	Effective	2	Medium
Ellis 2009	Non-randomised, pre-post survey, intervention vs. control	Australia	483	Mental health	↑<br>Pro bully attitude (*p* = < .05)<br>⟷<br>Pro victim attitude (NS)<br>↑<br>Honesty/trustworthiness: (*p* =< .05)<br>⟷<br>Self Confidence (NS)<br>⟷<br>Global self esteem (NS)<br>⟷<br>Self-efficacy (NS)<br>⟷<br>Same sex relations (NS)<br>↑<br>Opposite-sex relations (*p* = < .01)	Ineffective	4	High

					↔<br>Cooperative teamwork (NS)			
Kaveh et al. 2014	Cluster randomised controlled trial, intervention vs. control	Iran	223	Mental health	↑<br>Self-esteem general scale (*p*=<0.001)<br>↑<br>Academic scale (*p*=<0.001)<br>↑<br>Social scale (*p*=<0.001)<br>↔<br>Family scale (*p*=0.529)<br>↑<br>Body scale (*p*=<0.001)<br>↑<br>Total scale (*p*=<0.001)	Effective	3	Medium
Parikh et al. 2018	Non-randomised, pre-post survey	USA	878	Mental health	↑<br>Confidence in identifying peers with depression (*p*<.001)<br>↑<br>Confidence in referring peers with depression (*p* =<.001)<br>↔<br>Knowing whether to tell someone if a friend informs them they are suicidal (NS)<br>↑<br>Feelings about visiting school social worker/psychologist: (*p* = <.001)<br>↑<br>Depression knowledge questions: (*p* = <.001)<br>↑<br>Knowledge of depression symptoms: (*p* = <.001)<br>↔<br>Attitudes to a new student with depression: ns<br>↑<br>Comfort speaking to other students about mental health (*p* =< 0.001)	Effective	1	Low

					↑<br>Willingness to confide in authority figures<br>(*p* = <0.01)			
Aten et al. 2002	Non-randomised controlled trial, intervention vs. control	USA	1352	Sex education	Sexual activity at follow-up for students who were abstinent at pretest:<br>↔<br>Females: (*p* = 0.07)<br>↑<br>Males: (*p* = 0.04)	Mixed	3	Medium
Borgia et al. 2005	Randomised controlled trial, peer-led vs. teacher-led	Italy	1295	Sex education	↑↑↑↑<br>Pre-post: Skills, knowledge, attitudes, risk perception (p=<0.05)<br>↔↑↔↔<br>Between arms: Skills, knowledge, attitudes, risk perception (*p*=<0.05)	Effective	4	High
Caron et al. 2004	Non-randomised, intervention vs. control	Canada	1004	Sex education	↑<br>Postponing sexual intercourse: Intention (*p*=0.001)<br>↑<br>Postponing sexual intercourse: Attitude direct (*p*= < 0.001)<br>↑<br>Postponing sexual intercourse: Attitude indirect (*p*= < 0.001)<br>↑<br>Postponing sexual intercourse: Perceived self-efficacy (*p*= < 0.001)<br>↑<br>Postponing sexual intercourse: Role beliefs (*p*= < 0.001)<br>↑<br>Postponing sexual intercourse: Anticipated regret (*p* = < 0.001)<br>↑<br>Condom use: intention (*p* = < 0.001)	Effective	3	Medium

					↑<br>Condom use: roles beliefs (*p* = < 0.001)			
Fisher et al. 2002	Quasi-experimental controlled trial, classroom-based, peer-based and combined.	USA	1577	Sex education	**Information**<br>⟷<br>sexually inexperienced (NS)<br>↑<br>sexually experienced (*p*= < .01)<br><br>**Attitudes**<br>⟷<br>sexually inexperienced (NS)<br>↑<br>sexually experienced: (*p*= <.01)<br><br>**Norms**<br>⟷<br>sexually inexperienced (NS)<br>⟷<br>sexually experienced (NS)<br><br>Intentions<br>⟷<br>sexually inexperienced (NS)<br>⟷<br>sexually experienced (NS)<br><br>**Behavioural skills**<br>⟷<br>sexually inexperienced (NS)<br>⟷<br>sexually experienced (NS)	Ineffective	1	Low
Huang et al. 2008	Randomised, peer-led vs. teacher-led	China	3068	Sex education	↑<br>HIV-related knowledge (*p* =<0.001)<br>↑<br>Attitudes (*p* =<0.001)<br>↑<br>Behaviour intention (*p* =<0.05)	Effective	3	Medium

Jennings et al. 2014	Non-randomised intervention vs. control	USA	157	Sex education	↓<br>Knowledge (p=0.10)<br>↓<br>Parental communication (p=0.46)<br>↓<br>Self-efficacy (p=0.06)<br>↓<br>Sexual health information (p=0.06)<br>↑<br>Opportunity to practice skills (p=0.01)<br>↑<br>Intentions (p=0.04)	Ineffective	4	High
Mahat et al. 2010	Non-randomised, pre-post survey	USA	106	Sex education	↑<br>Mean score of total HIV/AIDS knowledge (*p* =< .001)<br>↑<br>Mean of the self-efficacy for limiting HIV risk behaviour score (*p* = < .001)	Effective	2	Medium
Mason-Jones, Mathews & Fisher 2011	Non-randomised, intervention vs. control	South Africa	2339	Sex education	↑<br>Not had sex (*p*=0.002)<br>⟷<br>First age sex (*p*=0.84)<br>⟷<br>Used a condom at last sex (*p*=0.32)<br>⟷<br>Goal orientation (*p*=0.20)<br>⟷<br>Decision-making (*p*=0.58)<br>⟷<br>Future orientation score (*p*=0.32)	Ineffective	3	High
Mellanby et al. 2001	Non-randomised, peer-led vs. adult-led	UK	1320	Sex education	↓<br>Knowledge of STDs (greater increase in adult-led, *p* = < 0.001)<br>⟷<br>Assertiveness score (*p* =0.32)<br>↑<br>Attitude scores (*p* =< 0.001)	Ineffective	3	Medium

					↓<br>Involvement in sessions (greater in adult-led, *p* = 0.016)<br>↓<br>Feeling embarrassed (greater in peer-led, *p* = <0.001)<br>↔<br>Talking to others about the sessions (*p*=0.12)			
Menna et al. 2015	Non-randomised, intervention vs. control	Ethiopia	560	Sex education	↑<br>Knowledge of HIV (*p*=0.004)<br>↔<br>Ever initiated sexual intercourse (*p*=0.72)<br>↔<br>Ever tested for HIV (0.53)<br>↑<br>Limiting sexual parter only to one in last 12 months (*p*=0.21)<br>↔<br>Consistent use of condom in last 12 months (*p*=0.24)<br>↑<br>Willingness to go for HIV counselling and testing (*p*=0.01)	Mixed	3	Medium
Merakou & Kourea-Kremastinou 2006	Non-randomised, intervention vs. control	Greece	702	Sex education	↑<br>Increased personal responsibility<br>↑<br>Safer behaviour in sexual practice<br>↔<br>Knowledge	Effective	1	Low
Michielsen et al. 2012	Non-randomised controlled trial, intervention vs. control	Rwanda	1400	Sex education	↔<br>Sexual risk behaviour (NS)<br>↔<br>Increased knowledge (NS)<br>↔<br>Perceived severity (NS)<br>↔<br>Perceived susceptibility (NS)	Ineffective	3	Medium

					↑<br>Stigma (*p*=0.05)			
Ozcebe, Akin & Aslan, 2004	Non-randomised, pre-post survey	Turkey	369	Sex education	↑<br>HIV/AIDS knowledge scores (*p* =<.001)	Effective	4	High
Parwej, 2005	Randomised, peer-led vs. nurse-led	India	273	Sex education	↑<br>Reproductive health domains<br>↑<br>Reproductive health knowledge<br>(*See full paper for full list of individual statements*)	Effective	3	Medium
Rotz et al. 2018	Matched comparison group design, intervention vs. comparison	USA	1522	Sex education	↔<br>Rates of sexual activity (*p*=.83)<br>↔<br>Rates of unprotected sex (*p*=.93)<br>↑<br>Exposure to information about sexual health topics (*p*=<.01)<br>↑<br>Knowledge of preventing pregnancy and transmission of STIs (p=<.01)	Mixed	3	Medium
Siegel et al. 1998	Non-randomised, intervention vs. control	USA	3635	Sex education	**Postintervention Knowledge Scores:**<br>↑<br>Middle School: Boys (*p* =<.001)<br>↑<br>Middle School: Girls (*p* =<.001)<br>↑<br>High School Boys (*p* =<.001)<br>↑<br>High School Girls (*p* =<.001)<br><br>**Self-efficacy Scores:**<br>↔<br>Middle School: Boys (NS)<br>↔<br>Middle School: Girls (NS)	Effective	3	Medium

					↑<br>High School Boys (*p* =<.001)<br>↑<br>High School Girls (*p* =<.001)<br><br>**Safe Behaviour Intention:**<br>↑<br>Middle School: Boys (*p* =<.001)<br>↑<br>Middle School: Girls (*p* =<.001)<br>↑<br>High School Boys (*p* =<.001)<br>↑<br>High School Girls (*p* =<.001)			
Siegel et al. 2001	Non-randomised, intervention vs. control	USA	4001	Sex education	**Postintervention Knowledge Scores:**<br>↑<br>Middle School: Boys (*p* =<.001)<br>↑<br>Middle School: Girls (*p* =<.001)<br>↑<br>High School Boys (*p* =<.001)<br>↑<br>High School Girls (*p* =<.001)<br><br>**Self-efficacy Scores:**<br>↔<br>Middle School: Boys (NS)<br>↑<br>Middle School: Girls (*p* =<.001)<br>↔<br>High School Boys (NS)<br>↑<br>High School Girls (*p* =<.001)<br><br>**Safe Behaviour Intention:**<br>↑<br>Middle School: Boys (*p* =<.001)	Mixed	3	Medium

					⟷<br>Middle School: Girls (NS)<br>⟷<br>High School Boys (NS)<br>⟷<br>High School Girls (NS)			
Stephenson et al. 2004	Cluster-randomised trial, intervention vs. control (teacher-led)	UK	9508	Sex education	↑<br>Girls: Unprotected sex before age 16 (*p*=0.0008)<br>⟷<br>Boys: Unprotected sex before age 16 (*p*=0·35)<br>↑<br>Girls: Knowledge of methods to prevent STIs (*p*=0.002)<br>⟷<br>Boys: Knowledge of methods to prevent STIs (NS)	Mixed	3	Medium
Stephenson et al. 2008	Cluster-randomised trial, intervention vs. control (teacher-led)	UK	9000	Sex education	⟷<br>Proportion of girls with one or more abortions by age 20 y (NS)<br>⟷<br>Proportion of girls who had had sex by age 18.0 y (NS)<br>⟷<br>Proportion of girls who had one or more live births by age 20.5 (NS)	Ineffective	3	Medium
Timol et al. 2016	Randomised, intervention vs. control	South Africa	2904	Sex education	↑<br>Sensation seeking (*p* =<.05)<br>↑<br>Self-efficacy in sexual relations (*p* =<.05)<br>↑<br>Future orientation (*p* =<.05)<br>↑<br>HIV transmission knowledge (*p* = <.01)<br>↑<br>HIV prevention knowledge (*p* =<.01)<br>↑<br>Health relationships (*p* = <.05)	Effective	3	Medium

Visser, 2007	Non-randomised, intervention vs. control	South Africa	4086	Sex education	⟷ Psychological wellbeing (NS)<br>⟷ Personal control (NS)<br>⟷ School climate (NS)	Ineffective	1	Low

↑ = improvement; ↓ = negative effect for peer education; ⟷ no effect

NS = no significant change where p-value is not reported

Effective = ≥60% outcomes with a positive finding; Mixed = 41-59% outcomes with a positive finding; Ineffective = ≥40% outcomes with a positive finding

High quality = 4-5; Medium quality = 2-3, Low quality = 0-1 (using the Mixed Methods Appraisal Tool, MMAT)

Supplementary Material 2: Full table of included study details, effectiveness and quality ratings for peer educator outcomes

Author, Year	Study Design	Nation	Sample Size	Health area	Outcome and effectiveness	Effectiveness	Quality Score	Quality Rating	Standalone paper?*
Audrey, Holliday & Campbell, 2006	Cluster randomised controlled trial, intervention vs. control	UK	10,730	Alcohol, smoking, substance use	Smoking prevalence at 1-year follow-up<br>↑<br>'High risk group' (*p* = 0.045)<br>⟷<br>Never smoked (*p* = 0.20)<br>⟷<br>Regular (weekly) smokers (*p*= 0.68)	Ineffective	5	High	Yes
Zambuto et al. 2019	Non-randomised, pre-post survey	Italy	524	Bullying	↑ ⟷<br>Victimization (*p*= .015 males, *p*= .093 females)<br>⟷ ⟷<br>Bullying (*p*= .068 males, *p*= .854 females)<br>↑⟷<br>Prosocial behaviour (*p*= .002 males, *p*= .455 females)<br>↑ ⟷<br>Social support (*p*= .036 males, *p*= .627 females)<br>⟷↑<br>Defending Behaviour(*p*= .925 males, *p*= .015 females)	Ineffective	3	Medium	Yes
Acemoglu, 2011	Non-randomised, pre-post survey	Turkey	2930	Disease prevention	↑<br>Knowledge scores (*p*=0.000)	Effective	3	Medium	No
Foley et al. 2017	Non-randomised, pre-post survey	Australia	519	Healthy lifestyles	↑<br>Eating ≥2 serves fruit/day (*p* =<0.01)<br>↑<br>Eating ≥5 serves vegetables/day	Mixed	4	High	Yes

					(*p* = <0.01)<br>⟷<br>Breakfast everyday (*p* = 0.053)<br>↑<br>Drinking <1 cup/day of Sugar sweetened beverages (*p* = <0.01)<br>⟷<br>Screen Time ≤ 2 h/day (*p* = 0.592)<br>⟷<br>Moderate-to-vigorous physical exercise ≥ 60 min/day (*p* =< 0.076)				
Eisenstein et al. 2019	Non-randomised, pre-post survey	UK	950	Mental health	↑<br>Understanding of key terms (*p* = 0.04)<br>↑<br>Key skills (*p* = <0.001)<br>⟷<br>Confidence to talk about mental health (*p* =0.08)<br>↑<br>Knowledge of information and resources (*p* = 0.02)<br>⟷<br>Readiness to support others (*p* =0.8)<br>⟷<br>Emotional difficulties (*p* = 0.24)<br>⟷<br>Behavioural difficulties (*p* =0.65)<br>⟷<br>School climate (*p* =0.5)	Ineffective	2	Medium	No
Parikh et al. 2018	Non-randomised, pre-post survey	India	878	Mental health	↑<br>Confidence in identifying peers with depression (*p* = 0.036)<br>↑<br>Confidence in referring peers with depression (*p*= 0.023)<br>↓	Ineffective	1	Low	No

					Knowing whether to tell someone if a friend informs them they are suicidal (NS)<br>↓<br>Feelings about visiting school social worker/psychologist (NS)<br>↓<br>Depression knowledge questions (NS)<br>↓<br>Knowledge of depression symptoms (*p* = 0.07)<br>↑<br>Attitudes to a new student with depression (NS)<br>↓<br>Comfort speaking to other students about mental health<br>(*p* = < 0.001)<br>↑<br>Willingness to confide in authority figures: peer educators: (NS)<br>↑				
Wyman et al. 2010	Randomised, intervention vs. wait-list control	USA	4128	Mental health	Help for suicidal peers (*p*=<.001)<br>↑<br>Rejects codes of silence (*p*=.002)<br>↑<br>Maladaptive coping (*p*=.013)<br>↑<br>Help-seeking from adults (*p*=<.001)<br>↑<br>Sources of Strength coping (*p*=.002)<br>↑<br>School engagement (*p*=.043)<br>↑<br>Trusted adults (*p*=<.001)<br>↑	Effective	4	High	Yes

					Referred distressed peers (*p*=.08)<br>⟷<br>Support to peers (*p*=.015)				
Caron et al. 2004	Non-randomised, intervention vs. control	Canada	1004	Sex education	↑<br>Postponing sexual intercourse: Attitude direct (*p* =< 0.001)<br>↑<br>Postponing sexual intercourse: Attitude indirect (*p* =< 0.001)<br>↑<br>Postponing sexual intercourse: Perceived behavioural control direct (*p* = < 0.001)<br>↑<br>Postponing sexual intercourse: personal normative beliefs (*p* =0.01)<br>↑<br>Postponing sexual intercourse: Role beliefs (*p* = < 0.001)<br>↑<br>Postponing sexual intercourse: Perceived self-efficacy (*p* = < 0.001)<br>↑<br>Postponing sexual intercourse: Anticipated regret (*p* =< 0.001)<br>↑<br>Condom use: behavioural control indirect (*p* =< 0.001)<br>↑<br>Condom use: perceived self-efficacy (*p* =< 0.001)<br>↑<br>Condom use: attitude direct measures (*p* =0.05)<br>↑<br>Condom use: attitude indirect measure	Effective	3	Medium	No

					(*p* =0.05)<br>↑<br>Condom use: personal normative beliefs (*p* =0.01)<br>↑<br>Condom use: anticipated regret (*p* =0.03)<br>↑<br>Condom use: perceived behavioural control direct measure (*p* =0.01)<br>↑<br>Condom use: role beliefs (*p* =0.05)<br>↑<br>Condom use: intention (*p* =0.02)				
Ebreo et al. 2002	Randomised, pre-post survey	USA	845	Sex education	↑<br>Had talked to their parents about the use of alcohol (*p* = < .05)<br>↓<br>Report having unwanted sex because they were intoxicated after the intervention (*p* =< .05) intervention inferior<br>⟷<br>Individual Difference Variables (NS)<br>⟷<br>Knowledge (NS)<br>⟷<br>Parental Communication (NS)<br>⟷<br>Peer Norms (NS)<br>⟷<br>Self-efficacy (NS)<br>⟷<br>Intentions to Have Sex (NS)<br>⟷<br>Self-reported behaviours (NS)<br>⟷	Ineffective	1	Low	Yes

					Student Course Evaluations (NS)				
Jennings et al. 2014	Non-randomised intervention vs. control	USA	160	Sex education	⟷<br>Knowledge (*p* =0.10)<br>⟷<br>Parental communication (*p*=0.46)<br>⟷<br>Self-efficacy (*p*=0.06)<br>⟷<br>Sexual health information (*p*=0.06)<br>↑<br>Opportunity to practice skills (*p*=0.01)<br>↑<br>Intentions (*p*=0.04)	Ineffective	4	High	No
Strange, Forrest & Oakley, 2002	Randomised controlled trial, intervention vs. control	UK	331	Sex education	⟷<br>Knowledge; condoms (*p* = 0.109)<br>⟷<br>Knowledge; the Pill (*p* =0.089)<br>↑<br>Knowledge; femidom (*p* =0.000)<br>↑<br>Knowledge; emergency contraception – (*p* =0.026)<br>↑<br>Knowledge; cap (*p* =0.000)<br>↑<br>Knowledge; coil (*p* =0.000)<br>⟷<br>Attitude; sex before marriage (*p* = 0.336)<br>↑<br>Attitude; people having sex with someone the first time they meet them: (*p* =0.002)<br>↑<br>Attitude; two men having sex with each other (*p* =0.01)<br>↑	Mixed	2	Medium	Yes

	Attitude; two women having sex with each other (p =0.003) ⟷ Attitude; abortion (p =0.7815) ⟷ Attitude; using contraception (p =0.2414)				

↑ = improvement; ↓ = negative effect for peer education; ⟷ no effect

NS = no significant change where p-value is not reported

Effective = ≥60% outcomes with a positive finding; Mixed = 41-59% outcomes with a positive finding; Ineffective = ≥40% outcomes with a positive finding

High quality = 4-5; Medium quality = 2-3, Low quality = 0-1 (using the Mixed Methods Appraisal Tool, MMAT)

**Standalone paper: a paper that reported peer educator outcomes only rather than peer learner and peer educator outcomes*

Supplementary Material 3: Example Search Strategy (Medline)

**Search terms pertaining to schools and school students**	**Search terms pertaining to peer education**	**Search terms pertaining to health-related interventions, health-related outcome domains**
(MH "Schools") OR TI ("Elementary School*" OR "Primary School*" OR "Junior School*" OR "Junior High School" OR "Secondary School*" OR "Middle School*" OR "Sixth Form*" OR "High School*" OR "Primary education" OR "Secondary education" OR "Special Education*" OR "School-based" OR "school based") OR AB ("Elementary School*" OR "Primary School*" OR "Junior School*" OR "Junior High School" OR "Secondary School*" OR "Middle School*" OR "Sixth Form*" OR "High School*" OR "Primary education" OR "Secondary education" OR "Special Education*" OR "School-based" OR "school based")	TI ("peer educat*" OR "peer-led" OR "Peer led" OR "Student-led" OR "Student led" OR "Peer teach*" OR "Peer Intervention* OR "Cross-age" or "Cross age" or "Peer to Peer" or "Peer-to-Peer" OR "Peer-delivered" or "Peer Delivered") OR AB ("peer educat*" OR "peer-led" OR "PEER LED" OR "STUDENT-LED" OR "STUDENT LED" OR "PEER TEACH*" OR "Peer Intervention* OR "Cross-age" or "Cross age" or "Peer to Peer" or "Peer-to-Peer" OR "Peer-delivered" or "Peer Delivered")	MH ("Health Education+" OR "Health Literacy+" OR “Health promotion” OR "School Health Services" OR "Preventive Health Services" OR “School Mental Health Services" OR "Anxiety" OR "Self Concept" OR "Depression” OR “Emotional Intelligence" OR “Psychological Distress” OR “Adaptation, Psychological" OR "Sex Education" OR "Sexual Behavior" OR "Safe Sex" OR "Tobacco Use" OR "Underage Drinking" OR "Alcoholism" OR "Substance-Related Disorders" OR "Substance Abuse, Intravenous” OR "Risk Reduction Behavior" OR "Impulsive Behavior" OR "Risk-Taking” OR “Health Risk Behaviors” OR "Health Behavior") OR TI (“Health Promotion" OR "Health Literacy" OR "health education" OR "School Based Intervention*" OR "Group Intervention*" OR “Smoking” OR “Drug abuse” OR “Substance abuse” OR “Drug education” OR "alcohol*" OR “Mental health” OR “Esteem” OR “Confidence” OR “Emotional” OR “behaviour*” OR “Depression” or “anxiety” OR “Sex education" OR "Safe sex" OR “RISK TAKING” OR “HEALTH RISK*”) OR AB ("Health Promotion" OR "Health Literacy" OR "health education" OR "School Based Intervention*" OR "Group Intervention*" OR “Smoking” OR “Drug abuse” OR “Substance abuse” OR “Drug education” OR "alcohol*" OR “Mental health” OR “Esteem” OR “Confidence” OR “Emotional” OR “behaviour*” OR “Depression” or “anxiety” OR “Sex education" OR "Safe sex" OR “RISK TAKING” OR “HEALTH RISK*”)
